# A Study on the Dual Thermo- and pH-Responsive Behaviors of Well-Defined Star-like Block Copolymers Synthesize by Combining of RAFT Polymerization and Thiol-Ene Click Reaction

**DOI:** 10.3390/polym14091695

**Published:** 2022-04-21

**Authors:** Yan Xue, Dan Huang, Xinyong Wang, Chunquan Zhang

**Affiliations:** Oil & Gas Field Applied Chemistry Key Laboratory of Sichuan Province, College of Chemistry and Chemical Engineering, Southwest Petroleum University, Chengdu 610500, China; 201921000236@stu.swpu.edu.cn (D.H.); xywang@swpu.edu.cn (X.W.); 201921000258@stu.swpu.edu.cn (C.Z.)

**Keywords:** reversible addition and fragmentation chain transfer (RAFT), star-like block copolymers, thiol-ene click reaction, dual-stimuli response

## Abstract

A series of stimuli-responsive star-like block copolymers are synthesized via the combination of reversible addition, fragmentation chain transfer (RAFT) polymerization, and photo-initiated thiol-ene (PITE) click reaction. The controllable block ratio and block sequence, narrow distribution of molecular weight, and customized arm numbers of the star-shaped copolymers reveal the feasibility and benefits of combination of RAFT polymerization and PITE click reaction for synthesis of well-defined star-like (co)polymers. A clear insight into the relationship among the arm number, block sequence, and block ratio of the star-like block copolymers and their stimuli-responsive aggregation behavior was achieved via dynamic light scattering and UV-vis spectroscopy study. Notably, the star-like poly(acrylic acid)-*b*-poly(2-(dimethylamino) ethyl methacrylate) (star-PAA-*b*-PDMAEMA) shows higher lower critical solution temperature (LCST) compared to star-PDMAEMA-*b*-PAA with the same arm number and block ratio due to the inner charged PAA segments at pH > IEP. In addition, for star-like PAA-*b*-PDMAEMA, higher PAA content enhances the hydrophilicity of the polymer in basic solution and leads to the LCST increase, except for star-PAA_1_-*b*-PDMAEMA_4_ at pH = 9.0 (≈IEP). For star-PDMAEMA-*b*-PAA, the PAA content shows minimal effect on their LCSTs, except for the polymer in solution with pH = 9.5, which is far from their IEP. The star-like block copolymers with well-defined structure and tunable composition, especially the facile-controlled block sequence, bring us a challenging opportunity to control the stimuli-responsive properties of star-like block copolymers.

## 1. Introduction

Star-like polymers generally refers to a type of polymer in which three or more linear polymer chains are connected to the same multifunctional group in the form of chemical bonds [1,2,3]. Compared with traditional chain polymers, star-like polymers have a series of special physical and chemical properties due to their special structure and three-dimensional shape, and have become one of the research hotspots in the polymer field. Stimuli-responsive star-like polymers introduced with stimuli-responsive groups have structural characteristics that are able to change sensitively with adjustment of the external environment, such as temperature, pH, light, and ionic strength, etc. [4,5,6]. To reach this end, incorporating stimuli-responsive amphiphilic block copolymers which exhibit “schizophrenic” micellization as “arms” is an alternative manner. To obtain relatively structured star architectures with narrow molecular weight distributions of “arms”, the controlled radical polymerization techniques (CRPTs) are usually implemented [7,8]. He et al. [5] used 2-Methoxyethyl acrylate (MEA), oligo(ethylene glycol) acrylate (OEG(M)A), and *tert*-butyl acrylate (*t*BA) as monomers to synthesize the pH- and thermo-stimuli responsive star-P*t*BA-*b*-P(MEA-*co*-OEGA_480_) with controllable molecular weight and narrow molecular weight distribution through photoinduced atom transfer radical polymerization (photo ATRP). However, metal catalysts were needed in the experiment, which easily polluted the environment. Therefore, RAFT polymerization without the metal catalyst has attracted the attention of scholars. It has been well acknowledged that RAFT polymerization, which functions by a degenerative chain transfer mechanism, exceeds other CRPTs in tolerance of functionalities and reaction conditions, and becomes the most versatile CRPT. Xu et al. [9] report a robust and efficient photoinduced living polymerization that enables monomer polymerization in the presence of air. The feature of this technology is that the thiocarbonylthio compound acts as an initiator in addition to the chain transfer agent in RAFT polymerization. Zhu et al. [10] describe an efficient photoinduced electron/energy transfer−reversible addition−fragmentation chain transfer (PET-RAFT) polymerization. Under the induction of light in the range of blue to red light (460 to 635 nm), PET-RAFT polymerization of various functional monomers was successfully carried out, and polymers with well-controlled molecular weight and narrow dispersion were obtained. Contributions by Rosselgong [11], Kobben [12], Herfurth [13], and Sun [14] have reported the precise synthesis of star-like polymers bonded 3~6 “arms” by RAFT polymerization. However, regarding the synthesis of non-polystyrene (PS) star-like polymers with a large number of “arms”, i.e., more than six “arms”, the advantages of RAFT severely discounted due to the significant termination reactions [15,16]. To address this limitation, we are enlightened by the grafting-onto approach of star-like polymer synthesis. Different from the well-established strategies of star-like polymer synthesis, i.e., arm-first and core-first approaches [17,18], the grafting-onto approach operates through a coupling reaction between the independently synthesized cores and arms. Therefore, high-level of structural control of star-like polymers could be achieved.

Since 2001, a highly efficient coupling strategy, i.e., click chemistry, has inspired researchers to apply it in a wide range of organic syntheses [19]. Wang et al. [20] successfully synthesized star-like polymers by the grafting-onto approach. Well-defined dibenzocyclooctyne(DIBO)-terminated styrene polystyrene (PS) linear and poly(ethylene glycol) (PEG) linear were prepared by atom transfer radical polymerization (ATRP) using a DIBO-containing ATRP initiator. Finally, the star-like polymers were successfully synthesized by strain-promoted azide-alkyne cycloaddition “click” reaction (SPAAC) with the azido tethered core. Modular click reactions are outstanding in quantitative yields with removable byproducts (if any) using non-chromatographic purification, mild reaction conditions with insensitivity to water or oxygen, and high tolerance of a wide variety of functional groups [21,22]. These characteristics also make click chemistry a promising methodology for highly functional and well-structured polymer synthesis [23,24]. Of special relevance is that the thiol-ene click reaction [25], one of the most important click reactions [26], can handily functionalize RAFT-synthesized polymers with more advanced architectures by cleavage of thiocarbonylthio end groups into thiols or thiolates [27]. In this vein, well-defined star-like polymers with controlled-structured arms can be obtained in high grafting efficiency. Zhang et al. [28] synthesized a variety of well-defined α, ω-telechelic polystyrenes with di- and tri-functionality through one-pot method combining the aminolysis of RAFT-polystyrene and the thiol-ene click reaction. Boyer et al. [29] developed a simple and catalyst-free method by combining thiol-ene click reaction and RAFT to prepare a series of ordered and controllable polymers with block sequence. On the basis of the prepared poly(ethylene glycol) (PEG), Sulistio et al. [30] successfully prepared the 36-arm star-like poly(PEG-*b*-L-lysine)_arm_ poly(L-cystine)_core_ with a lower molecular weight distribution through PITE “click” reaction. Combination of RAFT polymerization and thiol-ene click reaction has emerged as one of the most versatile and robust methods for preparing polymers with controlled molecular weight, molecular weight distribution, architectures, and functionality.

Concerning the stimuli that can trigger micellization behavior of responsive polymers, temperature and pH are two particularly important physiological parameters in our human environment. Thermo- and/or pH-responsive (co)polymers are the most extensively studied stimuli-responsive polymers, having important application values in controlled drug release and gene therapy [31,32,33]. Concerning the stimuli that can trigger micellization behavior of responsive polymers, temperature and pH are two particularly important physiological parameters in our human environment. Thermo- and/or pH-responsive (co)polymers are the most extensively studied stimuli-responsive polymers, having important application values in controlled drug release and gene therapy [31,32,33]. Winninger et al. [34] selected poly(ε-caprolactone) (PCL), poly(N-vinylcaprolactam) (PNVCL), and N-vinylpyrrolidone(NVP), and prepared a series of smart PCL_120_-*g*-PNVCL and PCL_120_-*g*-P(NVCL-*co*-NVP) graft copolymers. In aqueous media, these graft copolymers self-assemble into spherical micelles. When the polymerization degrees of the hydrophilic PNVCL grafted increases greatly, the micelle size of the copolymer also increases significantly. Furthermore, the addition of 10 or 20 mol% of NVP did not seem to change the micelle size. However, it results in a shift of the LCST of the amphiphilic graft copolymer to higher temperatures in the range of 38–40 °C. Atanase et al. [35] investigated the micellization behavior of pH-stimulable poly(2-vinylpyridine)-*b*-poly(ethylene oxide) (P2VP-*b*-PEO) copolymers. In the absence of the anionic surfactant sodium dodecylsulfate(SDS), the transition from protonated monomers to micelles occurs at pH around 5. For relatively high copolymer concentrations, the micellization range can be extended to lower pH values, whereas at low pH and low copolymer concentrations, only protonated monomers are present. However, the bihydrophilic copolymers form complexes through electrostatic interactions between SDS and protonated P2VP, so SDS can induce the polymer micellization. Poly(2-(dimethylamino) ethyl methacrylate) (PDMAEMA) is commonly used to synthesize thermo-responsive polymers. Its tertiary amine groups can be protonated in an acidic medium and are cationic at low pH values. It deprotonates the quaternized tertiary amine groups under alkaline conditions. This can induce the thermo-response of PDMAEMA to depend on pH. Shieh et al. [36] prepared a series of amphiphilic poly(2-(dimethylamino) ethyl methacrylate)-*b*-poly(*N*-isopropyl acrylamide) (PDMAEMA-*b*-PNIPAAm) diblock copolymers with dual CO_2_- and thermo-response; through the stimulation of specific conditions (temperature and CO_2_/N_2_ bubbling), they achieved effective control of the reversible emulsification process. Yuan et al. [37] prepared star-poly(caprolactone)-(poly(2-(dimethylamino) ethyl methacrylate)_7_) (star-PCL-(PDMAEMA)_7_) micellar solutions with unique temperature-fluorescence responsive behavior; through the transformation of the quaternization of the PDMAEMA segment in the star-like polymers, the lower critical solution temperature (LCST) of the solution was converted to upper critical solution temperature (UCST). Wang et al. [38] prepared a series of the amphiphilicity of 4-arm star-like poly(ε-caprolactone)-*b*-poly(2-(diethylamino)ethylmethacrylate) (4AS-PCL-*b*-PDMAEMA). Due to the pH-stimuli response, when the pH of the polymer solution was 5.0, the protonation of PDMAEMA accelerated the drug release rate of the polymer micelles. Hydrophilic poly (acrylic acid) (PAA) is an anionic polyelectrolyte, which is usually used to synthesize star-like electrolyte polymers. Iatridi et al. [39] successfully prepared the star-like (polystyrene)_22_(poly(2-vinylpyridine)-*b*-poly(acrylic acid))_22_, star-PS_22_(P2VP-*b*-PAA)_22_. Due to the weak polyamphodicity of P2VP-*b*-PAA diblock copolymer arms (positive ionization of P2VP, negative ionization of PAA), this star-like polymer shows pH-responsive properties. Enormous efforts have been devoted to the micellization of block polyampholytes and the responsiveness of corresponding star-like polymers, whereas the aggregation behavior of the star-like polyampholytes resulted from the stimuli-triggered “schizophrenic” micellization and the effect of block sequence have been rarely concerned. Our work focused on the thermo- and pH-responsive star-like PDMAEMA-*b*-PAA, of which the well-defined structure was constructed by combining RAFT polymerization and a PITE click reaction. The arms of the star-like polyampholytes composed of thermo- and pH-responsive PDMAEMA block and pH-responsive PAA block in a series of different proportions and molecular weight were synthesized by RAFT polymerization at first. Subsequently, conversion of each thiocarbonylthio end group into thiol group capable of grafting onto ene-capped dendritic cores was followed by PITE click reaction between RAFT-polymer arms and dendritic cores. Thanks to the excellent combination of RAFT and click technology, the structure and composition of the thermo- and pH-responsive star-like polymers can be well tuned, e.g., arm number, block proportion, block sequence, etc. The influence of the aforementioned elements of star-like polyampholyte design on the D*_h_* and LCST was investigated tuning the temperature and pH, indicating that the LCST and stimuli-responsive behavior of the star-like block copolymers can be effectively controlled by adjusting the star-like microstructure, especially the block sequence.

## 2. Materials and Methods

### 2.1. Materials

2-dimethylaminoethyl methacrylate (DMAEMA) (Aladdin, Shanghai, China, 99%) and *tert*-butyl acrylate (*t*BA) (Aladdin, 99%) were distilled under reduced pressure. Azodiisobutyronitrile (AIBN) (Aladdin, AR) was recrystallized from ethanol. Tetrahydrofuran (THF) (Adamas-beta, AR) and 1,4-dioxane (Aladdin, AR) were purified by standard methods before use. The dialysis bags (Spectrumlabs, Shanghai, China) with the molecular weight cutoff of 200~10,000 Da are used after being boiled for 20 min. Pentaerythritol (Adamas-beta, 98%), dipentaerythritol (Adamas-beta, 90%), trifluoroacetic acid (TFA) (Adamas-beta, 90%), 3-bromopropene (Adamas-beta, RG), ethyl ether (Adamas-beta, AR), sodium chloride (Adamas-beta, AR), magnesium sulfate (Aladdin, AR), sodium hydroxide (NaOH) (Aladdin, AR), 3-mercapto-1,2-propanediol (Adamas-beta, RG), 1-hydroxycyclohexyl phenyl ketone (photoinitiator 184) (Aladdin, 95%), and hydrazine hydrate (Adamas-beta, 98%) were used as received without further purification.

### 2.2. Preparation of 4-, 8-, and 12-Functionalized Cores via PITE Click Reaction

**Scheme 1:**
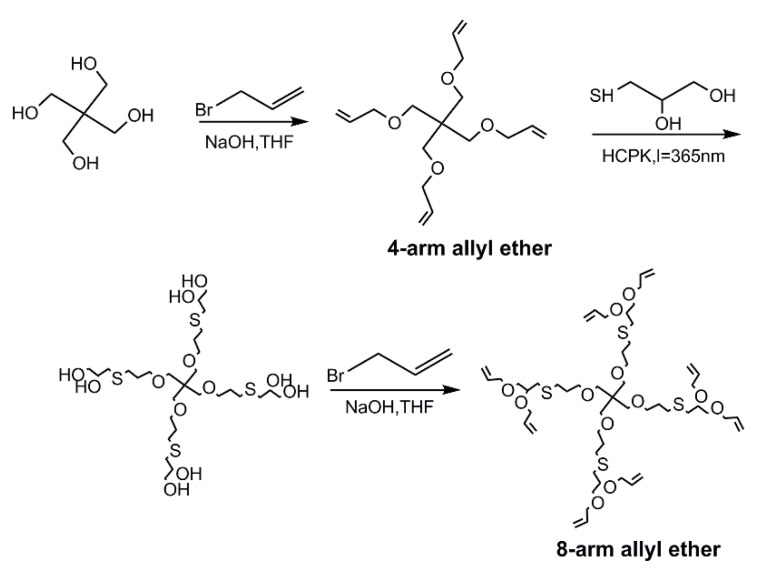
Preparation of 4-arm and 8-arm allyl ether.

The 4- and 8-functionalized cores, i.e., 4-arm and 8-arm allyl ether, of the star-like block copolymers were prepared following the procedure, as shown in Scheme 1. The 4-arm allyl ether was synthesized according to the literature [40]. Using 4-arm allyl ether as a precursor, 8-arm allyl ether was prepared by a PITE click reaction for arm multiplication.

Four-arm allyl ether (2.96 g, 10 mmol), 3-mercapto-1,2-propanediol (6.48 g, 60 mmol), and photoinitiator 184 (0.05 g, 0.13 mmol) were dissolved in THF (10 mL) under stirring at room temperature. The mixture was then purged with nitrogen for 30 min, sealed, and then irradiated for 2 h under an ultraviolet light (λ=365 nm). The product was precipitated and washed three times in ethyl acetate, followed by reduced pressure distillation. The obtained colorless, transparent and viscous liquid was then reacted with ally bromide under alkaline condition via a similar procedure for 4-arm allyl ether. ^1^H NMR of 8-arm allyl ether (400 MHz, CDCl_3_, Figure S2b): 1.80–1.84 (8H, -OCH_2_C***H***_2_CH_2_S-), 2.41–2.88 (16H, -C***H***_2_SC***H***_2_-), 3.35–3.78 (28H, -OC***H***_2_CH_2_CH_2_S-; -OC***H***_2_C***H***-; -OC***H***_2_C-), 3.89–4.18 (16H, -OC***H***_2_CH=CH_2_), 4.96–5.28 (16H, -OCH_2_CH=C***H***_2_), 5.75–5.93 (8H, -OCH_2_C***H***=CH_2_). Twelve-arm allyl ether was prepared following a similar procedure as 8-arm allyl ether, only dipentaerythritol was used instead of pentaerythritol.

### 2.3. Preparation of PDMAEMA-b-PtBA Block Copolymer Arms via RAFT Polymerization

**Scheme 2:**
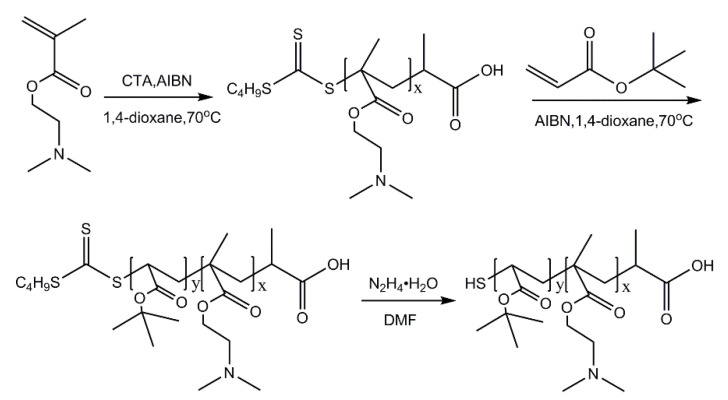
Synthetic route of HS-P*t*BA-*b*-PDMAEMA via RAFT polymerization.

The precursor of the block copolymer arms of the star-like block copolymers with outer PDMAEMA segments, i.e., HS-P*t*BA-*b*-PDMAEMA, was prepared via RAFT in three steps as shown in Scheme 2. A typical synthesis procedure of HS-P*t*BA-*b*-PDMAEMA is as follows. DMAEMA (1.00 g, 6.36 mmol), 2-{[(butylsul-fanyl) carbonothioyl]sulfanyl}propanoic acid as the chain transfer agent (CTA, 0.34 g, 1.43 mmol), AIBN (0.03 g, 0.18 mmol) and 1, 4-dioxane (6 mL) were added into a reaction tube with a stir bar. After degassing by three cycles of evacuate-argon-sparge, the RAFT polymerization was carried out in an oil bath at 70 °C. After 6 h, the polymerization was quenched by cooling the reaction tube to r.t. with exposure to air. The reaction solution was dialyzed against deionized water for three days and then freeze-dried to yield the product CTA-PDMAEMA as solid. CTA-P*t*BA-*b*-PDMAEMA was then prepared by a similar RAFT polymerization to CTA-PDMAEMA using the as-prepared CTA-PDMAEMA as the macro-CTA and *t*BA as the second comonomer. HS-P*t*BA-*b*-PDMAEMA was then prepared by thiol-modification of CTA-P*t*BA-*b*-PDMAEMA with the use of hydrazine hydrate. Other thiol-terminated block copolymers were prepared in a similar procedure with feed ratios, as listed in Tables S1 and S2.

### 2.4. Preparation of Star-like Block Copolymers via PITE Click Reaction

The star-like block copolymers were prepared via PITE click reaction. A typical procedure for the preparation of star-like PDMAEMA-*b*-P*t*BA block copolymers was as follows. Eight-arm allyl ether (0.24 g, 0.23 mmol), HS-P*t*BA_2_-*b*-PDMAEMA_4_ (2.12 g, 2.19 mmol), and the photoinitiator UV-184 (0.05 g, 0.13 mmol) were dissolved in DMF (6 mL). After three cycles of evacuate-argon-sparge, the mixture was UV-irradiated (λ=365 nm) under the protection of argon for 12 h at r.t. The reaction was quenched by exposure to air. Then the product was purified by dialysis against deionized water and followed by lyophilization to provide 8-arm star-P*t*BA_2_-*b*-PDMAEMA_4_. Eight-arm star-PAA_2_-*b*-PDMAEMA_4_ was obtained by specific hydrolysis of 8-arm star-P*t*BA_2_-*b*-PDMAEMA_4_ in trifluoroacetic acid (TFA). Other 4-, 8-, and 12-arm star-PAA-*b*-PDMAEMA and star-PDMAEMA-*b*-PAA with different block ratios were prepared following the similar procedure to 8-arm star-PAA_2_-*b*-PDMAEMA_4_ with the use of corresponding multi-arm allyl ether cores and thiol-terminated block copolymer arms.

### 2.5. Characterization

^1^H NMR spectra of chain polymers, star-like block copolymers and other products were recorded by the spectrometer instrument AVANCE III HD 400 M (Bruker, Fällanden, Switzerland). According to the solubility of these products, CDCl_3_ or D_2_O was selected as the solvent. These products were formulated into a solution with a concentration of 10.0 mg mL^−1^ and tested at room temperature. The apparent molecular weights and polydispersity index (PDI) of the polymers synthesized in each experimental stage were determined by GPC at 35 °C, using Rid-20A (Shimadzu, Long Beach, CA, USA). THF was used as the eluent with a flow rate of 1.0 mL min^−1^ and narrow dispersed polystyrenes (PS) were used as standard. The Zeta potential of the star-like block copolymers solution were measured by a Zeta PALS (Brookhaven, NY, USA) at 25 °C. The polymers solution with a concentration of 1.0 mg mL^−1^ were prepared by using ultrapure water, then adjusted to a series of solutions with different pHs and poured into a quartz cuvette with a size of 1 cm. Hydrodynamic diameters (D*_h_*) of the solution of star-like block copolymers were measured at 25 °C by using the dynamic light scattering instrument BI-200SM (Brookhaven, NY, USA). The scattering angle was set to 90°, the power of the laser light source was 150 W, and the wavelength of the incident laser was 532 nm. The star-like block copolymers were prepared into the solution with a concentration of 1.0 mg mL^−1^ and the samples were tested under acidic conditions (pH = 3) and alkaline conditions (pH = 9). The LCST of the star-like block copolymers solutions were measured in a UV–vis spectrophotometer (UV-1800, Shimadzu, Kyoto, Japan). The polymers solutions each having a concentration of 1.0 mg mL^−1^ and different pHs (range from 3 to 11) were prepared by using ultrapure water. At a wavelength of 500 nm, the transmittance of the star-like block copolymers was monitored in the quartz cuvette (1 cm width) as the functions of temperature. Heating and cooling scans were performed between 25 and 65 °C at a scanning rate of medium speed.

## 3. Results and Discussion

### 3.1. Preparation of Star-like Block Copolymers with Reverse-Ordered Blocks

**Scheme 3:**
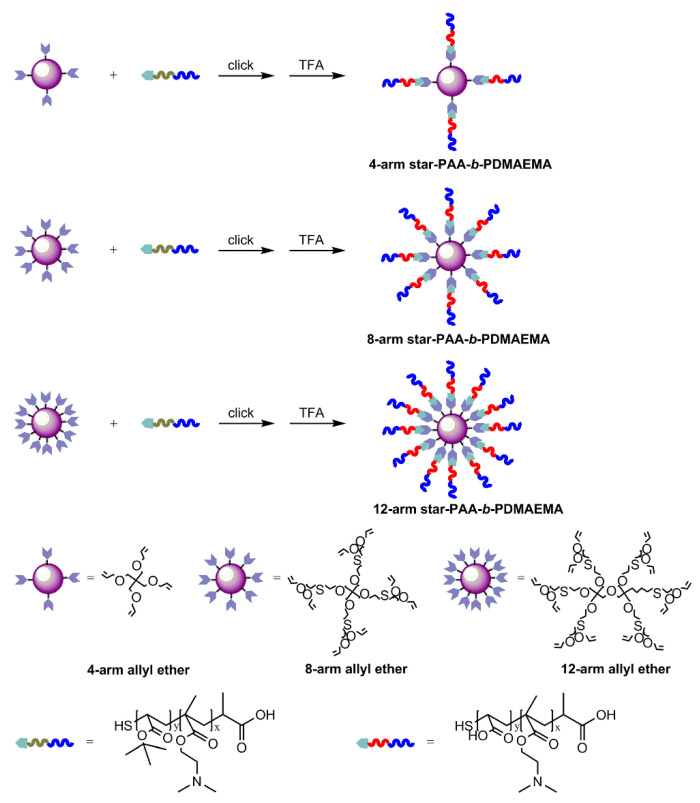
Preparation of 4-, 8-, and 12-arm star-like block copolymers with outer PDMAEMA segments.

**Figure 1:**
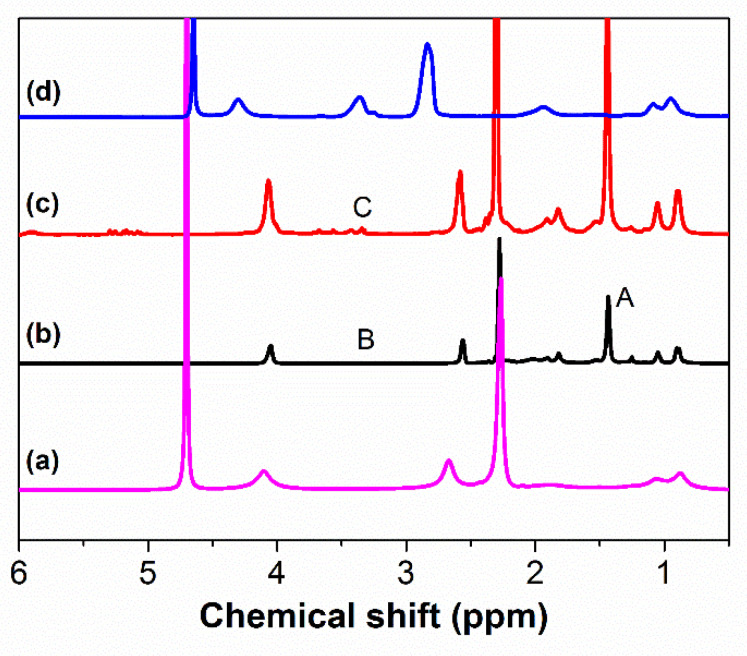
^1^H NMR spectra of CTA-PDMAEMA_4_ (**a**), HS-P*t*BA_2_-*b*- PDMAEMA_4_ (**b**), star-P*t*BA_2_-*b*-PDMAEMA_4_ (**c**) and star-PAA_2_-*b*-PDMAEMA_4_ (**d**).

As shown in Scheme 3, RAFT polymerization and PITE click chemistry were combined for synthesizing well-defined star-like block copolymers with controllable arm number, arm length, block ratio, and block sequence. To employ grafting-onto methodology, the star-like block copolymer cores with multiple end-allyl, i.e., 4-arm (yield = 72%), 8-arm (yield = 75%) and 12-arm (yield = 76%) allyl ether, were synthesized and have been characterized via 1H NMR spectra. (Figures S2 and S3). To prepare the block copolymer arms of the star-like block copolymers with reverse-ordered blocks and various block ratios, PDMAEMA or PtBA homopolymer was synthesized as the first block via RAFT polymerization, and then acted as the macro-CTA to generate the RAFT polymerization of the second comonomers, i.e., tBA or DMAEMA. The thiol-modification of the as-prepared macro-CTA was subsequently conducted using hydrazine hydrate. The example of star-PAA2-b-PDMAEMA4 with the arm precursor HS-PtBA2-b-PDMAEMA4 was selected to demonstrate the successful construction of star-like block copolymers by 1H NMR spectra. 1H NMR of CTA-PDMAEMA (400 MHz, CDCl_3_): 4.10 (2H, -OCH_2_CH_2_-), 2.67 (2H, -NCH_2_-CH_2_-), 2.48–2.05 (6H, -N(CH_3_)_2_), 1.85 (1H, -CCH_2_-), 0.96 (3H, -CCH_3_). 1H NMR of HS-PtBA-b-PDMAEMA (400 MHz, CDCl_3_, Figure S5): 0.96 (3H, -CCH_3_), 1.42 (9H, -C(CH_3_)_3_), 1.6–2.0 (3H, -CH_2_-; -CH-), 2.32 (6H, -N(CH_3_)_2_), 2.62 (2H, -OCH_2_CH_2_-), 4.10 (2H, -OCH_2_-). By comparing with spectrum Figure 1a of the macro-CTA PDMAEMA (denoted as CTA-PDMAEMA), the new peak (A peak) at 1.42 ppm in Figure 1b, assigned to -C(CH_3_)_3_ on the PtBA segment, indicates the successful block copolymerization of DMAEMA and tBA by sequence-RAFT polymerization. In addition, the absence of the peak assigned to the methylene proton on the CTA around δ = 3.38 ppm confirmed the successful thiol-termination on the block copolymers (Figure S5). As shown in Table 1 and Table S3, narrow molecular weight distributions of both the first block precursors (CTA-PDMAEMA or CTA-PtBA) and the block copolymers (within 1.04 and 1.16 respectively) were successfully achieved. In addition, the molecular weights and calculated block ratios of the RAFT-polymerized linear copolymers are close to the expected values.

Using UV-184 as the photoinitiator, the as-prepared block copolymer arms with thiol-end were grafted onto 4-arm, 8-arm, and 12-arm allyl ether via a PITE click reaction. Compared with the ^1^H NMR spectrum of HS-P*t*BA_2_-*b*-PDMAEMA_4_, the characteristic -OCH_2_C***H***_2_CH_2_S- peak (C peak) of 8-arm allyl ether was observed in the ^1^H NMR spectrum of star-P*t*BA_2_-*b*-PDMAEMA_4_ (Figure 1c), indicating that the HS-P*t*BA_2_-*b*-PDMAEMA_4_ arm had been successfully grafted onto the ally ether core by PITE click reaction. After TFA specific hydrolysis the P*t*BA segment in star-P*t*BA_2_-*b*-PDMAEMA_4_ was converted to PAA segment, which was determined by the complete disappearance of A peak in Figure 1d.

To elucidate star architectures of the copolymers synthesized by click reaction between thiol-modified arms and multi-arm ally ether cores, the arm number was calculated based on the GPC data of the resulting polymers. It is known that the molecular weight of branched polymers obtained from GPC deviates from corresponding theoretical values due to their different radius of gyration from linear polymers. The molecular weight of synthesized star-like block copolymers was therefore calibrated according to Zimm and Stocmayer’ calibration by introducing a shrinkage factor g (Equations (1) and (2)) [41].

(1) $$g=\left[\left(3f-2\right)/f^{2}\right]$$

(2) $$\frac{M_{n,\mathrm{app}}}{M_{n,\mathrm{star}}}=g^{0.43}$$

where  f is the number of arms of the star-like block copolymers. As shown in Table 1, the arm number of 4-arm, 8-arm, and 12-arm P*t*BA_2_-*b*-PDMAEMA_4_ calculated on their correction coefficients (1.22, 1.58, and 1.85) and calibrated molecular weight was 4.13, 7.24, and 10.87, respectively. Applying the same calibration, the calculated arm numbers of all the other star-like block copolymers with different block ratios and block sequence were listed in Table S4, suggesting the arm numbers of prepared star-like block copolymers were similar to the functionalities of their branched “cores”. It is worth noting that the PDI of all the star-like block copolymers, even the 12-arm star polymers, remains relatively low (within 1.32, Table 1 and Table S4). All these results suggest that compared to conventional “arm first” and “core first” methods, the PITE click reaction between RAFT-prepared (co)polymers and branched allyl cores is a highly efficient technology for the synthesis of well-defined star-like (co)polymers.

### 3.2. The Isoelectric Point (IEP) of the Multi-Arm Star-like Block Copolymers

**Figure 2:**
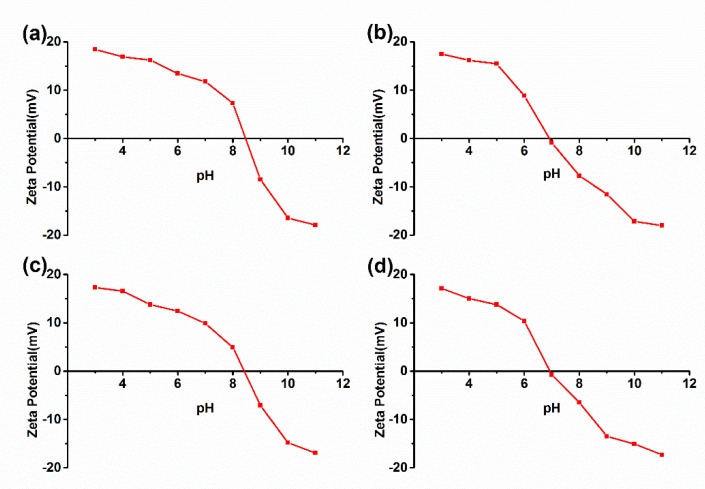
Zeta potential of 1.0 mg mL−^1^ solutions of 4-arm star-PAA_1_-*b*-PDMAEMA_4_ (**a**), star-PAA_2_-*b*-PDMAEMA_4_ (**b**), star-PDMAEMA_4_-*b*-PAA_1_ (**c**), and star-PDMAEMA_4_-*b*-PAA_2_ (**d**) at different pH values.

PDMAEMA is a representative LCST-type thermo-responsive polymer, of which the polymer solution becomes turbid above its LCST due to the phase change-induced precipitation of PDMAEMA. Nevertheless, the thermo-responsiveness or LCST of PDMAEMA is pH-dependent. The weak polybase PDMAEMA is water-soluble in acidic medium due to the protonation of the tertiary amine groups and shows no temperature-triggered turbidity. The quaternized tertiary amine groups of PDMAEMA, on the other hand, are deprotonated to a degree in basic medium which makes PDMAEMA exhibit temperature-dependent hydrophilicity/hydrophobicity transition. Furthermore, the incorporation of hydrophilic moiety into PDMAEMA can elevate its LCST while copolymerization of hydrophobic monomers with DMAEMA will lower its LCST. Herein, PAA segments displaying pH-dependent ionization and hydrophilicity were incorporated into star PDMAEMA by TFA-hydrolysis of P*t*BA block. Theoretically, the IEP of the star-like polyelectrolyte can be calculated using the following equation [42]:

(3) $$IEP=pK_{b}+\log\left\{\frac{1}{2}\left[\frac{1-R}{R}+{\left[{\left(\frac{1-R}{R}\right)}^{2}+\left(\frac{4}{R}\right)\times 10^{pK_{a}-pK_{b}}\right]}^{1/2}\right]\right\}$$

where R is the molar ratio of the acid to basic groups, and $pK_{a}$ and $pK_{b}$ are the dissociation constants of the acid and basic groups, respectively. The charge transition performance of polyampholytes is evidently related to the block ratio; therefore, the IEP of the star-like block copolymers with only four arms was determined using ζ-potential measurement (Figure 2). With two different block ratios and block orders, the star-PAA-*b*-PDMAEMA and star-PDMAEMA-*b*-PAA present IEP of 8.42 & 8.39 at R=1/4 and IEP of 6.87 & 6.92 at R=1/2. The theoretical isoelectric points are 8.48 and 8.00, respectively.

The experimental IEP is consistent with the calculated value of star-PAA-*b*-PDMAEMA with R=1/4 (IEP=8.48), whereas there is a deviation for the star-like block copolymers with R=1/2 (IEP=8.00). Furthermore, the above results indicate that the ζ-potential of the star-like block copolymers is hardly affected by the block sequence, which can be reasonably inferred that. When the pH value is close to IEP, the net charge of the star-like block polymer is almost zero, and hydrophobic interaction may cause aggregation and phase separation of the polymers. At lower or higher pH values, due to the electrostatic repulsion between a large number of net positive or negative charges distributed throughout the polymers, the star-like block copolymers can be dissolved in water, and no phase separation occurs.

### 3.3. pH- and Thermo-Responsive Behaviors of the Star-like Block Copolymer Solutions

Compared with linear hydrophobic/hydrophilic modified PDMAEMA, of which the pH and temperature dual-responsibility only depends on its block ratio, the star-like PAA-*b*-PDMAEMAs exhibit more complicated dual-responsibility which may be affected by their arm number, arm length, block ratio, and block order, etc.

#### 3.3.1. pH-Responsive Self-Assembly of Star-like PAA-b-PDMAEMA

**Figure 3:**
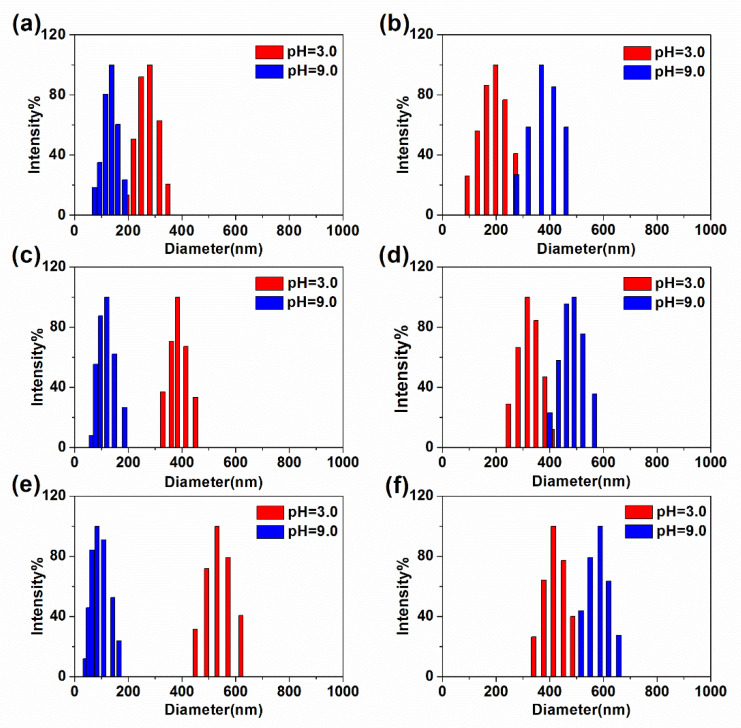
The apparent *D*_h_ distributions of 1.0 mg mL^−1^ aqueous solutions of 4-arm star-PAA_2_-*b*-PDMAEMA_4_ (**a**), 4-arm star-PDMAEMA_4_-*b*-PAA_2_ (**b**), 8-arm star-PAA_2_-*b*-PDMAEMA_4_ (**c**), 8-arm star-PDMAEMA_4_-*b*-PAA_2_ (**d**), 12-arm star-PAA_2_-*b*-PDMAEMA_4_ (**e**), 12-arm star-PDMAEMA_4_-*b*-PAA_2_ (**f**).

Irrespective of the effect of temperature on the hydrophilicity/hydrophobility of the star-like PAA-*b*-PDMAEMAs, their pH-responsive self-assembly behavior was firstly investigated via DLS measurements at ambient temperature, i.e., ~20 °C. D*_h_* distributions of star-like PAA_2_-*b*-PDMAEMA_4_ and PDMAEMA_4_-*b*-PAA_2_ with different arm numbers under acidic (pH = 3.0) and alkaline (pH = 9.0) conditions were shown in Figure 3.

All the star-like block copolymers display unimodal D*_h_* distribution with the peak > 80 nm, indicating all of them formed micelle aggregates under the testing conditions. It is known that the dissociation of PAA is inhibited in acidic solutions, and meanwhile PDMAEMA is positively charged due to protonation of the tertiary amine groups. At pH = 3.0 the inner PAA segment of star-PAA_2_-*b*-PDMAEMA_4_ shrinks into a nucleus while the outer PDMAEMA segment stretches into a charged shell, forming aggregates with D*_h_* of 280, 388 and 535 nm, respectively (Figure 3a,c,e). In comparison, the peaks of D*_h_* distribution of the star-like block copolymers with same arm number but reverse ordered blocks have no apparent shift under the same pH condition (Figure 3b,d,f), presumably due to the short outer PAA segments (only half of the length of PDMAEMA segments) exerting little effect on the inner protonated PDMAEMA arms. However, increasing pH above the IEP of the star-like block copolymers (pH = 9.0) leads to distinctly different D*_h_* distributions of star-like PAA-*b*-PDMAEMAs with reverse block orders. Under alkaline conditions, the small negatively-charged nucleus formed by inner dissociated PAA segments interfere with the aggregate formation of star-like block copolymers, showing D*_h_* of star-PAA_2_-*b*-PDMAEMA_4_ smaller than 135 nm (Figure 3a,c,e). On the contrary, the outer ionic PAA segments extend the inner deprotonated quaternized PDMAEMA segments, promoting the formation of large aggregates of star- PDMAEMA_4_-*b*-PAA_2_, for all of which the D*_h_* distributions are larger than 376 nm (Figure 3b,d,f). The sequential order of D*_h_* distributions of star-like block copolymers at pH = 3.0 and pH = 9.0 overturns as their block orders reverse in essence. Moreover, in most cases, aggregate size of star-like block copolymers increases with arm number increasing when the pH of the polymer solution is constant, since increasing arm number is propitious for micelle aggregating. Except for star-PAA-*b*-PDMAEMA under alkaline conditions, its aggregate size decreases as the number of arms increases. In this regard, the star-PAA-*b*-PDMAEMA is in a hydrophobic state as a whole. When the number of arms of the star-like block copolymers increases, the degree of shrinkage of star-PAA-*b*-PDMAEMA will increase, and the micellar core will become more compact. This results in a reduction in aggregate size.

#### 3.3.2. The Effect of Block Sequence and Arm Number on pH-Tuned Thermo-Responsive Behaviors of Star-like Block Copolymers

**Figure 4:**
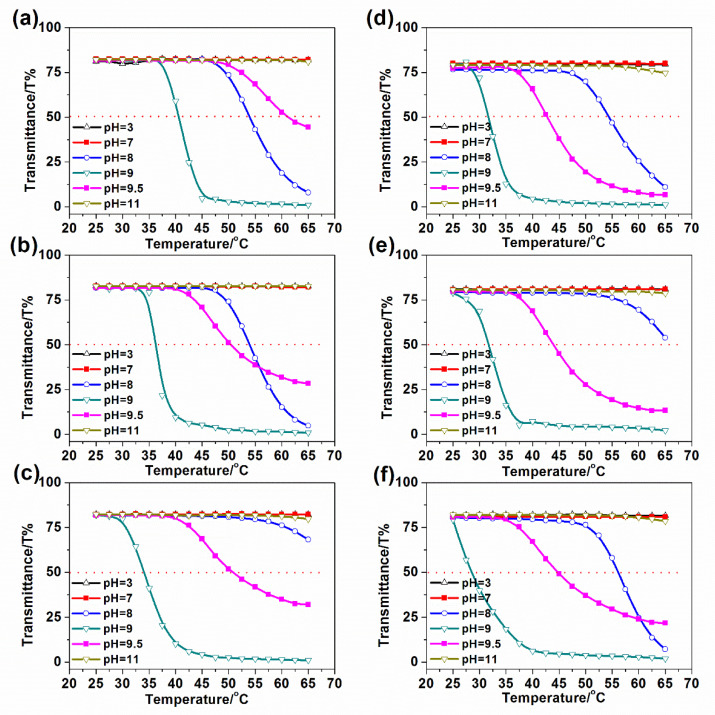
The Effect of the pH value of 1.0 mg mL^−1^ aqueous solutions of 4-arm star-PAA_1_-*b*-PDMAEMA_4_ (**a**), 8-arm star-PAA_1_-*b*-PDMAEMA_4_ (**b**), 12-arm star-PAA_1_-*b*-PDMAEMA_4_ (**c**), 4-arm star-PDMAEMA_4_-*b*-PAA_1_ (**d**), 8-arm star-PDMAEMA_4_-*b*-PAA_1_ (**e**), 12-arm star-PDMAEMA_4_-*b*-PAA_1_ (**f**) on the LCST and temperature curve when the transmittance of the solution was monitored at 500 nm at a heating rate of 0.5 °C min^−1^.

The thermo-responsive behaviors of star-PAA_1_-*b*-PDMAEMA_4_ and star-PDMAEMA_4_-*b*-PAA_1_ with different arm numbers in solutions at a series of pH are shown in Figure 4.

At low pH, the PDMAEMA segments are protonated and fully stretched, accordingly preventing the hydrophilic star-like block copolymers from temperature-triggered phase separating. Therefore, there is no LCST detected for all the test polymer solutions at pH < IEP, i.e., pH = 3.0 & 7.0. It should be pointed out that the star-like block copolymers could form micelle aggregates as mentioned above, which results in the transmittance of polymer solutions lower than 100% even at ambient temperature. At a high pH, like pH ≥ 11.0, the quaternized PDMAEMA segments are deprotonated while the PAA segments are completely ionized. The fully ionized PAA segments can interfere with the hydrophobic association of PDMAEMA segments and consequently hamper the phase separation of the star-like block copolymer solutions. The results indicate that for the star-like block copolymers with the block ratio of PAA to PDMAEMA of 1:4, when the pH value of the polymer solution is far from the IEP, it will not exhibit obvious thermo-responsiveness. In this vein, the strong electrostatic repulsion resulted from completely protonated PDMAEMA segments or ionized PAA segments play a dominant role regardless of the arm number or block sequence. However, when the pH value of the polymer solutions is near IEP, the transmittance of the star-like block copolymer solution fluctuated significantly with the change of temperature and exhibited different variation depending on the arm number and block sequence.

**Figure 5:**
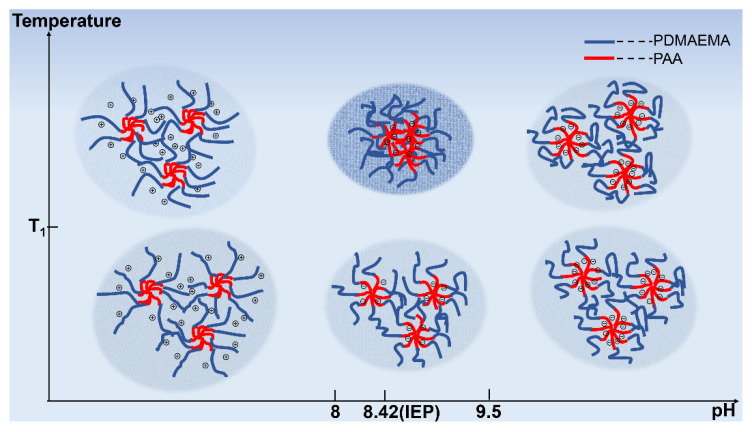
Schematic diagram of the pH- and thermo-response of the star-like PAA_1_-*b*-PDMAEMA_4_ solution.

**Figure 6:**
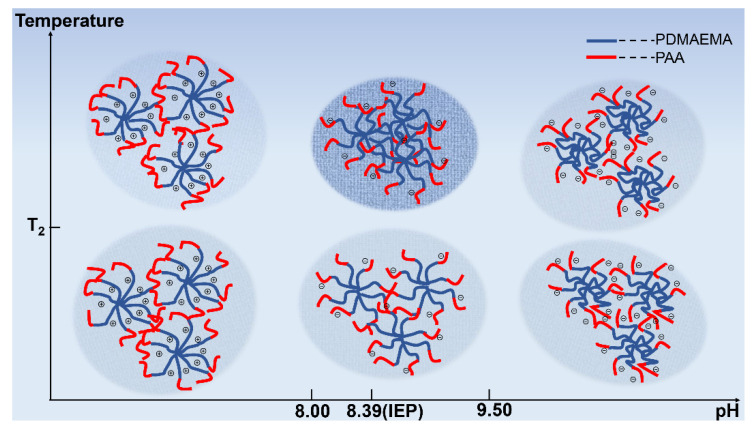
Schematic diagram of the pH- and thermo-response of the star-like PDMAEMA_4_-*b*-PAA_1_ solution.

Figure 4 demonstrates that at pH = 9.0 and pH = 9.5, the LCST of the star-like PDMAEMA-*b*-PAA solution is generally lower than that of the star-like PAA-*b*-PDMAEMA solution with the same arm number (Figure 5 and Figure 6, T_1_ in Figure 5 is larger than T_2_ in Figure 6). Although the star-like block copolymers as a whole is hydrophilic, the thermo-response of the polymer solution is dominant because of the longer PDMAEMA segments than PAA. As mentioned in Section 3.3.1, the star-like block copolymers with PAA outer segments are more likely to form compact aggregates in basic solution due to the collapsed inner PDMAEMA segments. Consequently, the star-like PDMAEMA-*b*-PAA solution is comparatively easier to experience phase separation induced by temperature-triggered hydrophobic association. The LCST of the star-like block copolymers at pH = 8.0 is generally higher than the LCST at pH = 9.0 and pH = 9.5. It is presumably due to the charging of both PAA and PDMAEMA segments around IEP, shifting the phase transition to higher temperature. In addition, the LCST of the star-like block copolymers with the same block sequence and the block ratio of PAA to PDMAEMA of 1:4 decreases as the number of arms increases. As shown in Figure 4, the LCST of 4-arm, 8-arm, and 12-arm star-like PAA_1_-*b*-PDMAEMA_4_ were approximately 41 °C, 36 °C, and 34 °C, respectively. The LCST of 4-arm, 8-arm, and 12-arm star-like PDMAEMA_4_-*b*-PAA_1_ were approximately 34 °C, 32 °C, and 29 °C, respectively.

The arm number effect on the thermo-response of the star-like block copolymers is resulted from the stronger hydrophobic association of the star-like block copolymers with a larger number of arms. The star-like block copolymers with more arms are more sensitive to temperature and unstable in water, and therefore prone to aggregate.

#### 3.3.3. The Effect of Block Sequence and Arm Number on pH-Tuned Thermo-Responsive Behaviors of Star-like Block Copolymers

**Figure 7:**
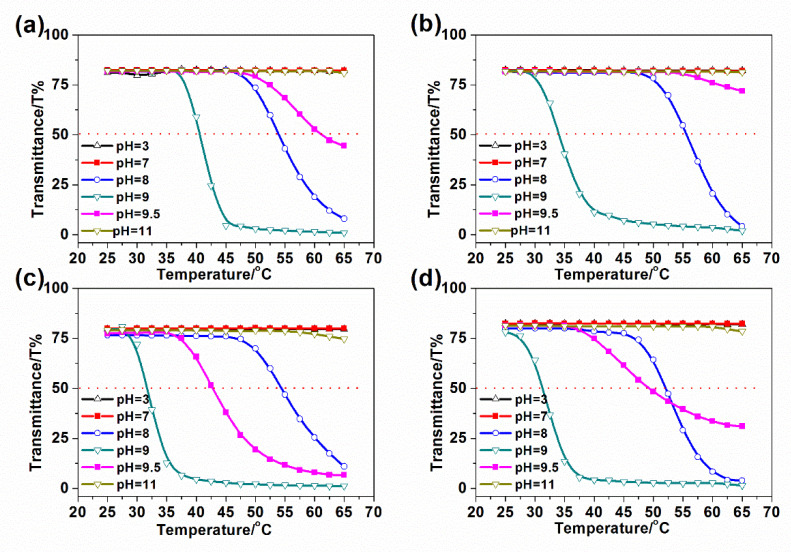
The effect of the pH value of 1.0 mg mL^−1^ aqueous solutions of 4-arm star-PAA_1_-*b*-PDMAEMA_4_ (**a**), 4-arm star-PAA_2_-*b*-PDMAEMA_4_ (**b**), 4-arm star-PDMAEMA_4_-*b*-PAA_1_ (**c**) and 4-arm star-PDMAEMA_4_-*b*-PAA_2_ (**d**) on the LCST and temperature curve when the transmittance of the solution was monitored at 500 nm at a heating rate of 0.5 °C min^−1^.

In addition to the number of arms and block sequence of the star-like block copolymers having a significant effect on the stimuli-responsive behaviors of the polymers, the block ratio also presents an effect on their stimuli-responsive behaviors. For 4-arm star-PAA-*b*-PDMAEMA and star-PDMAEMA-*b*-PAA, at pH 8.0, 9.0, and 9.5, the LCST of polymer solutions showed significant thermo-response, which was similar to the rule found above (Figure 7). However, the effect of PAA content on the thermo-responsive performance of the star-like block copolymers is discrepant due to their different block sequence. For star-PAA-*b*-PDMAEMA in basic solution, the inner ionized PAA segments hamper the hydrophobic association of the outer PDMAEMA segments. The LCST of 4-arm star-PAA_2_-*b*-PDMAEMA_4_ with higher block ratio of PAA/PDMAEMA is higher than that of 4-arm star-PAA_1_-*b*-PDMAEMA_4_ at pH = 8.0 and pH = 9.5. At pH = 9.0 (close to the IEP of star-PAA_1_-*b*-PDMAEMA_4_), the LCST of 4-arm star-PAA_1_-*b*-PDMAEMA_4_ was abnormally higher than that of 4-arm star-PAA_2_-*b*-PDMAEMA_4_. Both PAA and PDMAEMA segments are charged, making the hydrophilicity of the 4-arm star-PAA_1_-*b*-PDMAEMA_4_ enhanced, shifting the phase transition to higher temperatures. For star-like PDMAEMA-*b*-PAA, although the star-like block copolymers as a whole is hydrophilic, the thermo-response of the polymer solution is dominant because of the longer PDMAEMA segments than PAA. For 4-arm star-PDMAEMA-*b*-PAA with the outer negatively-charged PAA segments, the length of the PAA segments shows minimal effect on their LCSTs at pH = 8.0 and pH = 9.0. However, at pH = 9.5 which is far from the IEP of both 4-arm star-PDMAEMA_4_-*b*-PAA_1_ and 4-arm star-PDMAEMA_4_-*b*-PAA_2_, the latter polymer solution is more difficult to experience temperature-induced phase separation than the former one due to the enhanced hydrophilicity. In addition, there are similar rules found for 8-arm and 12-arm star-like block copolymers (Figures S10 and S11).

## 4. Conclusions

Chain block copolymers with controllable chain length and block ratio were successfully prepared by RAFT reaction. A series of stimuli-responsive star-like block copolymers with different arm numbers, block sequence, block ratios, and arm length were successfully synthesized via the combination of RAFT polymerization and a PITE click reaction. Applying sequence-RAFT polymerization between AA and DMAEMA and subsequent thiol-modification, narrow molecular weight distributions of thiol-terminated block copolymer chains were obtained with expected molecular weights and block ratios. The PITE click reaction between the RAFT-polymerized block copolymer arms and multi-functionalized ally ether cores efficiently afforded well-defined 4-, 8-, and 12-arm star-like block copolymers with reverse-ordered blocks. The effect of arm number, block sequence, and block ratio on the stimuli-responsive aggregation behavior of the as-prepared star-like block copolymers was investigated by dynamic light scattering and UV-vis spectroscopy. All the star-like block copolymers present thermo-responsiveness in aqueous solution, expect for pH ≤ 7.0 or pH ≥ 11.0. Generally, the LCST of the star-like block copolymers with the same block sequence decreases as the number of arms increases because of the enhanced hydrophobic association, as expected. It is intriguing that the block sequence plays a dominant role in affecting the aggregation behavior and corresponding stimuli-responsive performance of the star-like block copolymers. The star-like block copolymers with inner PAA segments are more difficult to form micellar aggregation and have higher LCST compared to star-PDMAEMA-*b*-PAA with the same arm number and block ratio, since the inner ionized PAA segments interfere with the hydrophobic effect of the outer PDMAEMA segments in basic solution. In addition, the block sequence also makes a difference to the effect of block ratio on the stimuli-responsiveness of the star-like block copolymers. For star-PAA-*b*-PDMAEMA, the higher PAA content enhances the hydrophilicity of the polymer in basic solution and leads to the LCST increase, except for star- PAA-*b*-PDMAEMA at pH = 9.0, which is closer to the IEP of star-PAA_1_-*b*-PDMAEMA_4_. For star-PDMAEMA-*b*-PAA, the PAA content shows minimal effect on their LCSTs except for the polymer in solution with pH = 9.5 which is far from the IEP of both star-PDMAEMA_4_-*b*-PAA_1_ and star-PDMAEMA_4_-*b*-PAA_2_. The results indicate that the block sequence, besides arm number and block ratio, is also a significant factor for tuning the stimuli-responsive behavior of star-like block copolymers, and thus provides a promising controlled pattern for star-like block copolymers in the application of drug and/or gene carriers.

## Figures and Tables

**Table 1:** Molecular weights and PDI of chain polymers and star-like block copolymers.

Sample	*M*_n,th_^(a)^ (g mol ^−1^)	*M*_n,app_^(b)^ or *M*_n,star_^(c)^ (g/mol)	PDI ^(b)^	n (DMAEMA)/ n (*t*BA) ^(d)^	Arm Number ^(c)^
CTA-PDMAEMA_4_	835	864	1.04	-	-
HS-P*t*BA_2_-*b*-PDMAEMA_4_	965	1138	1.03	2.09	-
4-Star-P*t*BA_2_-*b*-PDMAEMA_4_	4256	4999	1.23	2.09	4.13
8-Star-PtBA_2_-*b*-PDMAEMA_4_	8877	9282	1.30	2.09	7.24
12-Star-PtBA_2_-*b*-PDMAEMA_4_	12,547	13,997	1.18	2.09	10.87

^(a)^ Calculated by monomer conversion. ^(b)^ Determined by GPC. ^(c)^ Determined by GPC and Equations (1) and (2). ^(d)^ Evaluated Determined from ^1^H NMR. $M_{n,\mathrm{th}}$ is the theoretical molecular weight of chain polymers and star-like block copolymers. $M_{n,\mathrm{app}}$ is the apparent molecular weight measured by GPC, and $M_{n,\mathrm{star}}$ is the actual molecular weight of the star-like block copolymers.

## Data Availability

The data presented in this study are available on request from authors Y.X. and D.H.

## Supplementary Materials

The following supporting information can be downloaded at: https://www.mdpi.com/article/10.3390/polym14091695/s1, Figure S1: 1H-NMR spectrum of 2-(((butylthio)carbonothioyl)thio)propanoic acid in CDCl3; Figure S2: 1H NMR spectroscopy of 4-arm allyl ether (a) and 8-arm allyl ether (b) in CDCl3; Figure S3: 1H NMR spectroscopy of 6-arm allyl ether (a) and 12-arm allyl ether (b) in CDCl3; Figure S4: 1H NMR spectroscopy of CTA-PtBA; Figure S5: 1H-NMR spectrum of CTA-PtBA-b-PDMAEMA in CDCl3; Figure S6: 1H NMR spectroscopy of HS-PtBA1-b-PDMAEMA4 (a), HS-PtBA2-b-PDMAEMA4 (b), HS-PDMAEMA4-b-PtBA1 (c), HS-PDMAEMA4-b-PtBA2 (d); Figure S7: 1H NMR spectroscopy (400 MHz) of star-PAA1-b-PDMAEMA4 (a) and star-PAA2-b-PDMAEMA4; Figure S8: GPC traces of chain polymer; Figure S9: GPC traces of star-like block copolymers; Figure S10: The Effect of the pH value of 1.0 mg mL-1 aqueous solutions of 8-arm star-PAA1-b-PDMAEMA4 (a), 8-arm star-PAA2-b-PDMAEMA4 (b), 8-arm star-PDMAEMA4-b-PAA1 (c) and 8-arm star-PDMAEMA4-b-PAA2(d) on the LCST and temperature curve when the transmittance of the solution was monitored at 500 nm at a heating rate of 0.5oC min^−1^; Figure S11: The Effect of the pH value of 1.0 mg mL-1 aqueous solutions of 12-arm star-PAA1-b-PDMAEMA4 (a), 12-arm star-PAA2-b-PDMAEMA4 (b), 12-arm star-PDMAEMA4-b-PAA1(c) and 12-arm star-PDMAEMA4-b-PAA2 (d) on the LCST and temperature curve when the transmittance of the solution was monitored at 500 nm at a heating rate of 0.5oC min^−1^; Figure S12: Temperature dependences of the UV -visible transmittance of 1.0 mg mL-1 aqueous solutions at pH = 9.0 of 4-arm star-PAA2-b-PDMAEMA4 (a), 8-arm star-PAA2-b-PDMAEMA4 (b), 12-arm star-PAA2-b-PDMAEMA4 (c), 4-arm star-PDMAEMA4-b-PAA2 (d), 8-arm star-PDMAEMA4-b-PAA2 (e), 12-arm star-PDMAEMA4-b-PAA2 (f) on the LCST and temperature curve when the transmittance of the solution was monitored at 500 nm at a heating rate and cooling rate of 0.5oC min^−1^; Table S1: Preparation of CTA-PDMAEMA and CTA-PtBA; Table S2: Preparation of CTA-PtBA-b-PDMAEMA and CTA-PDMAEMA-b-PtBA; Table S3: Compositions, molecular weights and molecular-weight distributions of liner polymers; Table S4: Compositions, molecular weights and molecular-weight distributions of star-like block copolymers. Reference [43] are cited in the supplementary materials.

## References

[1] Guo Y., Li M., Li X., Shang Y., Liu H. (2017). Stimuli-responsive and micellar behaviors of star-shaped poly[2-(dimethylamino)ethyl methacrylate]-b-poly[2-(2-methoxyethoxy)ethyl methacrylate] with a β-cyclodextrin core. React. Funct. Polym..

[2] Wang J., Wang X., Yang F., Shen H., You Y., Wu D. (2014). Self-Assembly Behavior of a Linear-Star Supramolecular Amphiphile Based on Host–Guest Complexation. Langmuir.

[3] Zhang H., Yan Q., Kang Y., Zhou L., Zhou H., Yuan J., Wu S. (2012). Fabrication of thermo-responsive hydrogels from star-shaped copolymer with a biocompatible β-cyclodextrin core. Polymer.

[4] Sun H., Kabb C.P., Sims M.B., Sumerlin B.S. (2019). Architecture-transformable polymers: Reshaping the future of stimuli-responsive polymers. Prog. Polym. Sci..

[5] He J., Zhang W., Lv C., Chen R., Wang L., Wang Y., Pan X. (2021). Thermo- and pH-responsive star-like polymers synthesized by photoATRP. Polymer.

[6] Jana S., Uchman M. (2020). Poly(2-oxazoline)-based stimulus-responsive (Co)polymers: An overview of their design, solution properties, surface-chemistries and applications. Prog. Polym. Sci..

[7] Grubbs R.B., Grubbs R.H. (2017). 50th Anniversary Perspective: Living Polymerization—Emphasizing the Molecule in Macromolecules. Macromolecules.

[8] Braunecker W., Matyjaszewski K. (2008). Erratum to: “Controlled/living radical polymerization: Features, developments and perspectives”. Prog. Polym. Sci..

[9] Xu J., Jung K., Atme A., Shanmugam S., Boyer C. (2014). A Robust and Versatile Photoinduced Living Polymerization of Conjugated and Unconjugated Monomers and Its Oxygen Tolerance. J. Am. Chem. Soc..

[10] Zhu Y., Liu Y., Miller K.A., Zhu H., Egap E. (2020). Lead Halide Perovskite Nanocrystals as Photocatalysts for PET-RAFT Polymerization under Visible and Near-Infrared Irradiation. ACS Macro Lett..

[11] Rosselgong J., Williams E.G.L., Le T.P., Grusche F., Hinton T.M., Tizard M., Gunatillake P., Thang S.H. (2013). Core Degradable Star RAFT Polymers: Synthesis, Polymerization, and Degradation Studies. Macromolecules.

[12] Kobben S., Ethirajan A., Junkers T. (2014). Synthesis of Degradable Poly (methyl methacrylate) Star Polymers via RAFT Copolymerization with Cyclic Ketene Acetals. J. Polym. Sci. Part A Polym. Chem..

[13] Herfurth C., Laschewsky A., Noirez L., von Lospichl B., Gradzielski M. (2016). Thermoresponsive (star) block copolymers from one-pot sequential RAFT polymerizations and their self-assembly in aqueous solution. Polymer.

[14] Sun Z., Wang M., Li Z., Choi B., Mulder R.J., Feng A., Moad G., Thang S.H. (2020). Versatile Approach for Preparing PVC-Based Mikto-Arm Star Additives Based on RAFT Polymerization. Macromolecules.

[15] Boschmann D., Vana P. (2007). Z-RAFT Star Polymerizations of Acrylates:  Star Coupling via Intermolecular Chain Transfer to Polymer. Macromolecules.

[16] Zhang C., Zhou Y., Liu Q., Li S., Perrier S., Zhao Y. (2011). Facile Synthesis of Hyperbranched and Star-Shaped Polymers by RAFT Polymerization Based on a Polymerizable Trithiocarbonate. Macromolecules.

[17] Cakir Yigit N., Tunca U., Hizal G., Durmaz H. (2016). Heterofunctionalized Multiarm Star Polymers via Sequential Thiol- para -Fluoro and Thiol-Ene Double “Click” Reactions. Macromol. Chem. Phys..

[18] Ren J.M., McKenzie T.G., Fu Q., Wong E.H.H., Xu J., An Z., Shanmugam S., Davis T.P., Boyer C., Qiao G.G. (2016). Star Polymers. Chem. Rev..

[19] Chen Y., Xianyu Y., Wu J., Yin B., Jiang X. (2016). Click Chemistry-Mediated Nanosensors for Biochemical Assays. Theranostics.

[20] Wang S.S., Yang X.K., Zhu W., Zou L., Zhang K., Chen Y.M., Xi F. (2014). Strain-promoted azide-alkyne cycloaddition “click” as a conjugation tool for building topological polymers. Polymer.

[21] Hoyle C.E., Bowman C.N. (2010). Thiol–Ene Click Chemistry. Angew. Chem. Int. Ed..

[22] Fan P.-R., Zhao X., Wei Z.-H., Huang Y.-P., Liu Z.-S. (2020). Robust immobilized enzyme reactor based on trimethylolpropane trimethacrylate organic monolithic matrix through “thiol-ene” click reaction. Eur. Polym. J..

[23] Sitterli A., Heinze T. (2019). Studies about reactive ene-functionalized dextran derivatives for Thiol-ene click reactions. React. Funct. Polym..

[24] Winkler R., Pellet-Rostaing S., Arrachart G. (2020). Efficient and multi-function compatible click-reaction of organosilanes. Tetrahedron Lett..

[25] Shen X., Liu P., He C., Xia S., Liu J., Cheng F., Suo H., Zhao Y., Chen L. (2021). Surface PEGylation of polyacrylonitrile membrane via thiol-ene click chemistry for efficient separation of oil-in-water emulsions. Sep. Purif. Technol..

[26] Kade M.J., Burke D.J., Hawker C.J. (2010). The power of thiol-ene chemistry. J. Polym. Sci. Part A Polym. Chem..

[27] Harvison M.A., Lowe A.B.J.M.R.C. (2011). Combining RAFT Radical Polymerization and Click/Highly Efficient Coupling Chemistries: A Powerful Strategy for the Preparation of Novel Materials. Macromol. Rapid Commun..

[28] Zhang S., Cui K., Huang J., Zhao Q., Cao S. (2015). Synthesis of Diverse α,ω-Telechelic Polystyrenes with Di- and Tri-functionality via Tandem or One-Pot Strategies Combining Aminolysis of RAFT-Polystyrene and Thiol-Ene “Click” Reaction. RSC Adv..

[29] Fu C., Huang Z., Hawker C., Moad G., Xu J., Boyer C. (2017). RAFT-mediated, visible light-initiated single unit monomer insertion and its application in the synthesis of sequence-defined polymers. Polym. Chem..

[30] Sulistio A., Lowenthal J., Blencowe A., Bongiovanni M.N., Ong L., Gras S.L., Zhang X., Qiao G.G. (2011). Folic Acid Conjugated Amino Acid-Based Star Polymers for Active Targeting of Cancer Cells. Biomacromolecules.

[31] Chen L., Feng W., Zhou X., Yin Z., He C. (2015). Thermo-and pH dual-responsive mesoporous silica nanoparticles for controlled drug release. J. Control. Release.

[32] Patil A., Gadad A. (2018). Development and Evaluation of High Oxaliplatin Loaded CS-g-PNIPAAm Co-Polymeric Nanoparticles for Thermo and pH Responsive Delivery. Indian J. Pharm. Educ. Res..

[33] Hooshyar Z., Bardajee G. (2016). A novel dual thermo- and pH-responsive silver nanocomposite hydrogel as a drug delivery system. J. Iran. Chem. Soc..

[34] Winninger J., Iurea D.M., Atanase L.I., Salhi S., Delaite C., Riess G. (2019). Micellization of novel biocompatible thermo-sensitive graft copolymers based on poly(ε-caprolactone), poly(N-vinylcaprolactam) and poly(N-vinylpyrrolidone). Eur. Polym. J..

[35] Atanase L.I., Riess G. (2013). Micellization of pH-stimulable poly(2-vinylpyridine)-b-poly(ethylene oxide) copolymers and their complexation with anionic surfactants. J. Colloid Interface Sci..

[36] Shieh Y.-T., Tai P.-Y., Cheng C.-C. (2019). Dual CO_2_/Temperature-Responsive Diblock Copolymers Confer Controlled Reversible Emulsion Behavior. Polym. Chem..

[37] Yuan W., Zou H., Guo K., Wang A., Ren J. (2012). Supramolecular amphiphilic star-branched copolymer: From LCST-UCST transition to temperature-fluorescence responses. J. Mater. Chem..

[38] Yu C., Wang L., Xu Z., Teng W., Wu Z.-M., Xiong D. (2020). Smart micelles self-assembled from four-arm star polymers as potential drug carriers for pH-triggered DOX release. J. Polym. Res..

[39] Iatridi Z., Tsitsilianis C. (2013). pH responsive MWCNT–star terpolymer nanohybrids. Soft Matter.

[40] Quick A.S., Fischer J., Richter B., Pauloehrl T., Trouillet V., Wegener M., Barner-Kowollik C. (2013). Preparation of Reactive Three-Dimensional Microstructures via Direct Laser Writing and Thiol-ene Chemistry. Macromol. Rapid Commun..

[41] Zimm B., Stockmayer W. (1949). The Dimensions of Chain Molecules Containing Branches and Rings. J. Chem. Phys..

[42] Sakai K., Vamvakaki M., Smith E.G., Wanless E.J., Armes S.P., Biggs S. (2008). Adsorption characteristics of zwitterionic diblock copolymers at the silica/aqueous solution interface. J. Colloid Interface Sci..

[43] Ferguson C.J., Hughes R.J., Smith E.G., Pham B.T.T., Hawkett B.S., Gilbert R.G., Serelis A.K., Such C.H. (2002). Effective ab Initio Emulsion Polymerization under RAFT Control. Macromolecules.

