# Supplementary material for: A Study on the Dual Thermo- and pH-Responsive Behaviors of Well-Defined Star-like Block Copolymers Synthesize by Combining of RAFT Polymerization and Thiol-Ene Click Reaction
Source: Polymers (Basel). 2022 Apr 21;14(9):1695. doi: 10.3390/polym14091695 (PMC9103776; doi:10.3390/polym14091695)
Supplement: Supplementary file 1 [file polymers-14-01695-s001.zip › polymers-1684034-supplementary.pdf]

Supporting Information

A Study on the Dual Thermo- and pH-Responsive Behaviors of Well-Defined Star-Like Block Copolymers Synthesize by Combining of RAFT Polymerization and Thiol-Ene Click Reaction

Synthesis of the RAFT agent

2-(((butylthio)carbonothioyl)thio)propanoic acid (BCTPA) was selected as the RAFT chain transfer agent (CAT)[1].

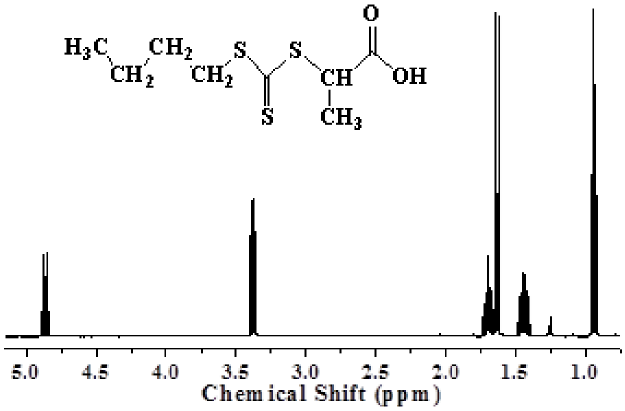

Figure S1. ^{1}H-NMR spectrum of 2-(((butylthio)carbonothioyl)thio)propanoic acid in $CDCl_3$.

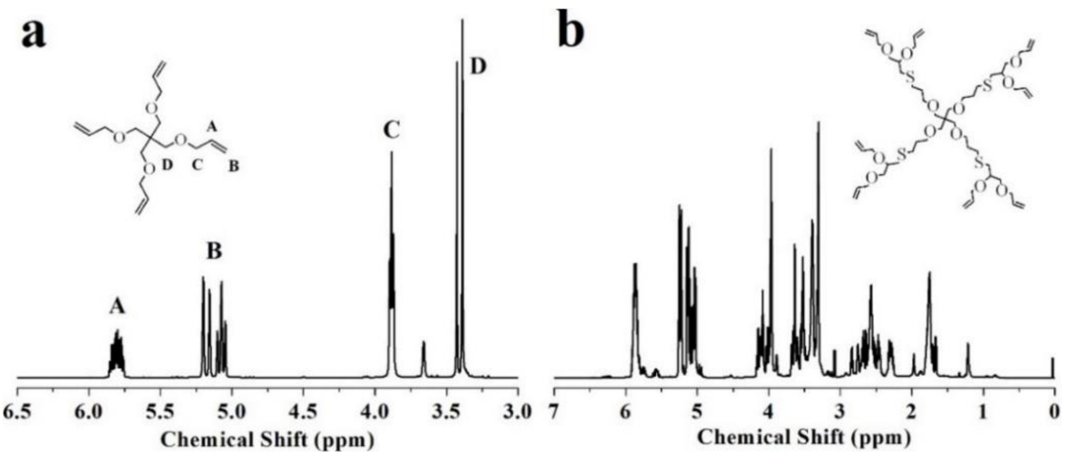

Figure. S2. ^{1}H NMR spectroscopy of 4-arm allyl ether (a) and 8-arm allyl ether (b) in $CDCl_3$.

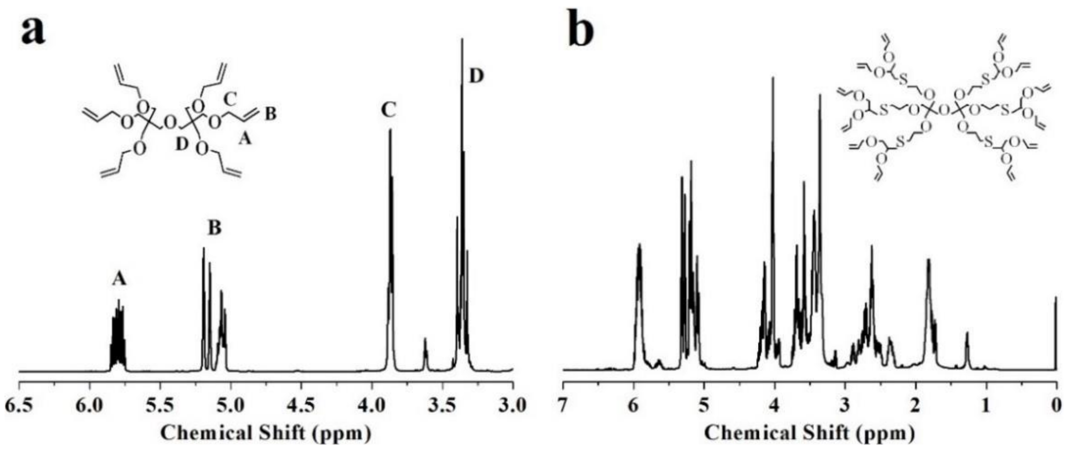

Figure. S3. ^{1}H NMR spectroscopy of 6-arm allyl ether (a) and 12-arm allyl ether (b) in $CDCl_3$.

Table S1. Preparation of CTA-PDMAEMA and CTA-P*t*BA

sample	Monomer (g)		CTA (g)	AIBN (g)
	DMAEMA	*t*BA		
CTA-$PDMAEMA_4$	1.00	-	0.38	0.02
CTA-$PDMAEMA_8$	1.00	-	0.19	0.01
CTA-P*t*BA_1	-	0.32	0.60	0.04
CTA-P*t*BA_2	-	0.32	0.30	0.02
CTA-P*t*BA_4	-	0.32	0.15	0.01

Table S2. Preparation of CTA-P*t*BA-*b*-PDMAEMA and CTA-PDMAEMA-*b*-P*t*BA

sample	macro-CTA (g)		Monomer (g)		AIBN (g)
	CTA-PDMAEMA	CTA-P*t*BA	PDMAEMA	*t*BA	
CTA-P*t*BA_1-*b*-$PDMAEMA_4$	1.00	-	-	0.15	0.02
CTA-P*t*BA_2-*b*-$PDMAEMA_8$	1.00	-	-	0.15	0.01
CTA-P*t*BA_4-*b*-$PDMAEMA_8$	1.00	-	-	0.30	0.01
CTA-$PDMAEMA_4$-*b*-P*t*BA_1	-	0.50	0.90	-	0.02
CTA-$PDMAEMA_4$-*b*-P*t*BA_2	-	0.50	0.61	-	0.02
CTA-$PDMAEMA_8$-*b*-P*t*BA_2	-	0.50	1.22	-	0.02
CTA-$PDMAEMA_8$-*b*-P*t*BA_4	-	0.50	0.72	-	0.01

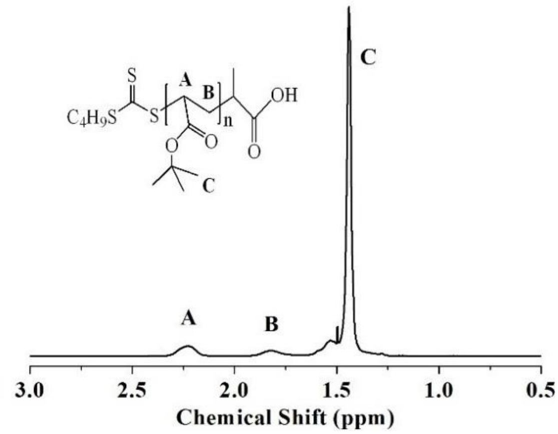

Figure S4. ^{1}H NMR spectroscopy of CTA-P*t*BA.

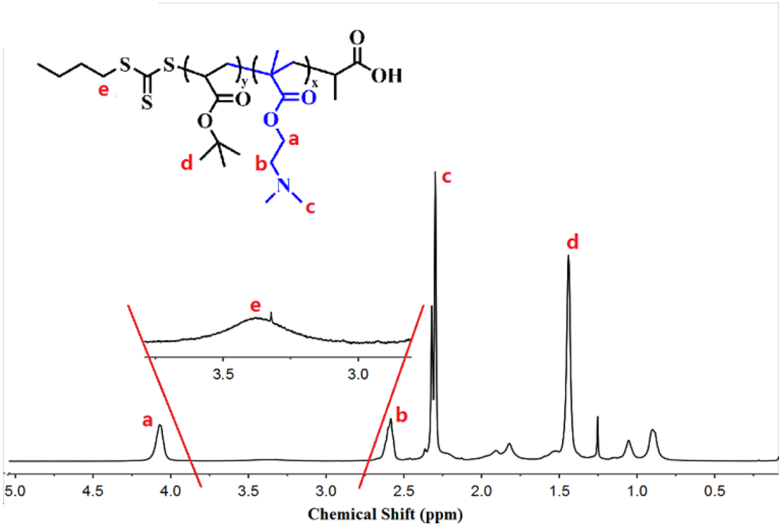

Figure S5. ^{1}H-NMR spectrum of CTA-P*t*BA-*b*-PDMAEMA in $CDCl_3$.

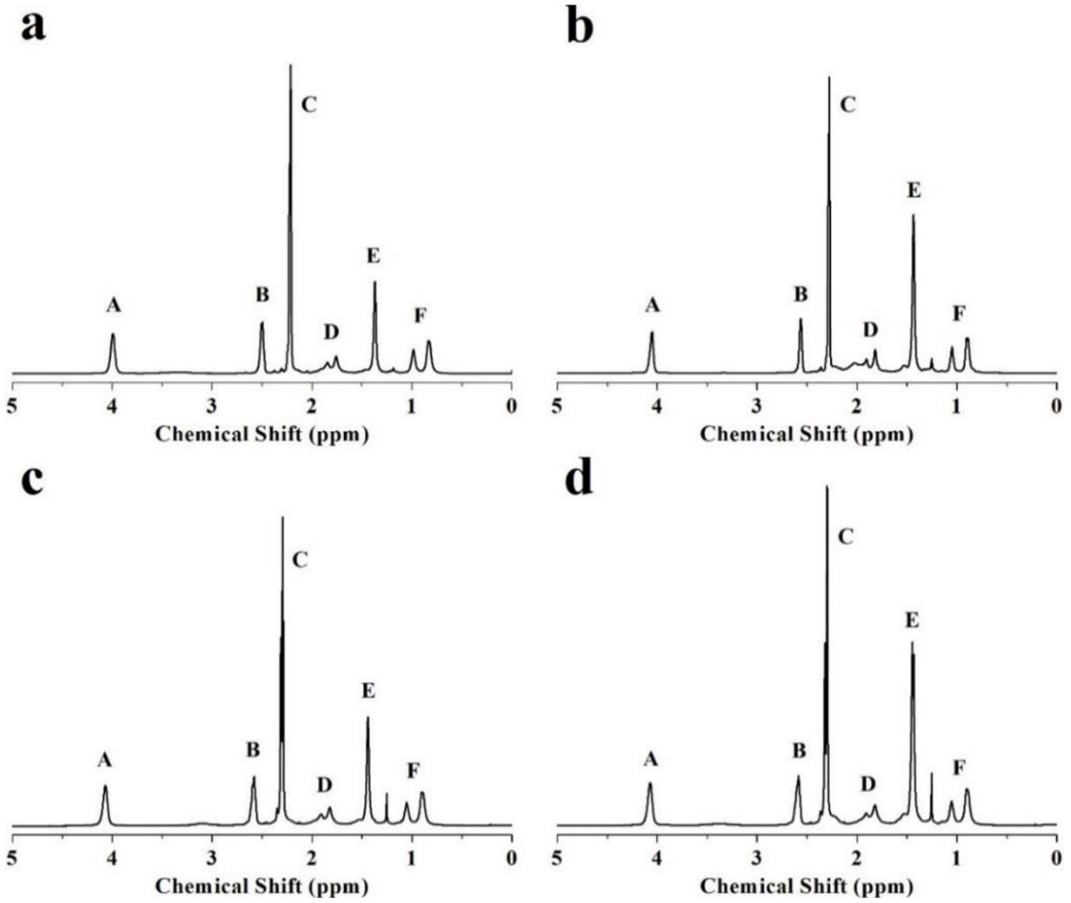

Figure S6. ^{1}H NMR spectroscopy of HS-P*t*BA$_1$-*b*-PDMAEMA$_4$ (a), HS-P*t*BA$_2$-*b*-PDMAEMA$_4$ (b), HS-PDMAEMA$_4$-*b*-P*t*BA$_1$ (c), HS-PDMAEMA$_4$-*b*-P*t*BA$_2$ (d).

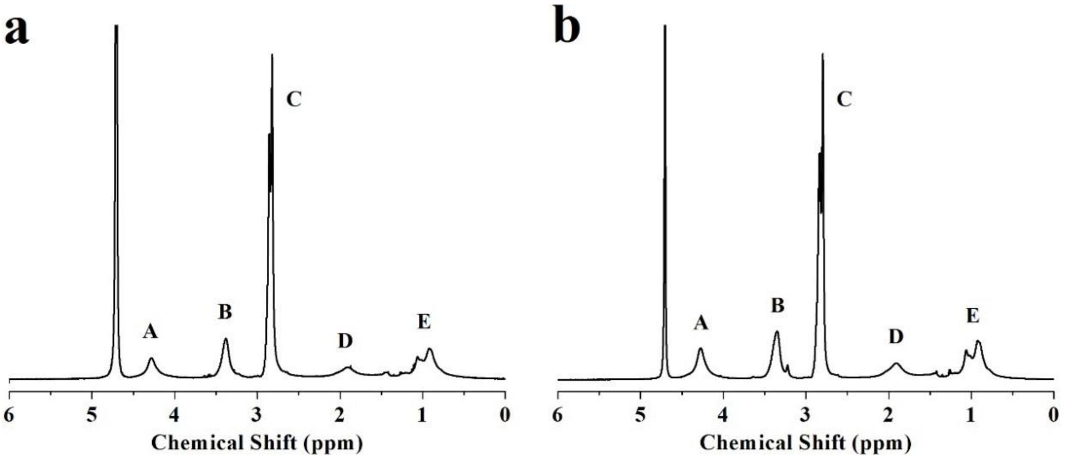

Figure. S7. ^{1}H NMR spectroscopy (400 MHz) of star-PAA_1-*b*-$PDMAEMA_4$ (a) and star-PAA_2-*b*-$PDMAEMA_4$

.

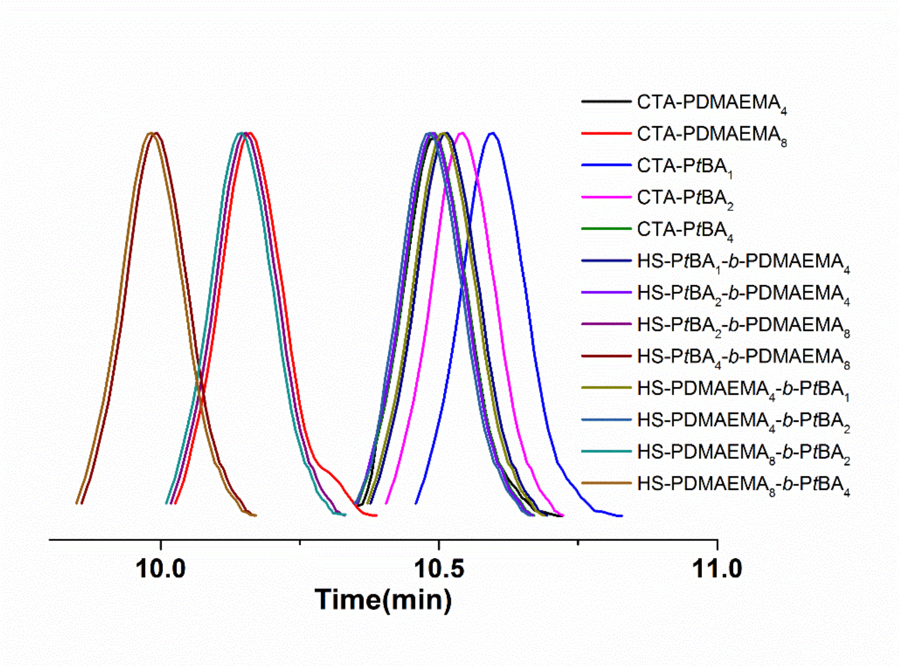

Figure S8. GPC traces of chain polymer.

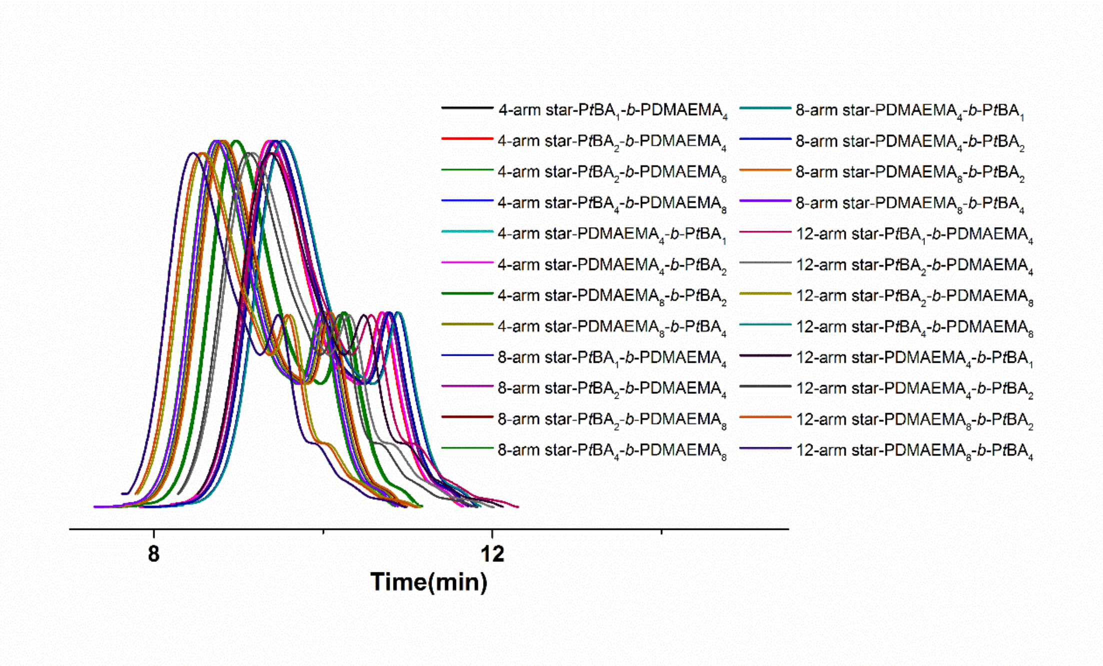

Figure S9. GPC traces of star-like block copolymers.

Table S3. Compositions, molecular weights and molecular-weight distributions of liner polymers.

Sample	Conversion [a] (%)	$M_{n,th}$ [b] (g·mol^{-1})	n(DMAEMA)/n(*t*BA) [a]	$M_{n,app}$ [c]	PDI [c]
CTA-$PDMAEMA_8$	93	1408	-	1712	1.04
CTA-P*t*BA_1	90	353	-	348	1.01
CTA-P*t*BA_2	92	474	-	517	1.03
CTA-P*t*BA_4	89	695	-	876	1.04
HS-P*t*BA_1-*b*-$PDMAEMA_4$	89	846	4.47	913	1.07
HS-P*t*BA_2-*b*-$PDMAEMA_8$	92	1816	5.09	1918	1.14
HS-P*t*BA_4-*b*-$PDMAEMA_8$	94	2062	2.49	2257	1.16
HS-$PDMAEMA_4$-*b*- P*t*BA_1	91	788	4.23	935	1.14
HS-$PDMAEMA_4$-*b*- P*t*BA_2	95	982	1.75	1050	1.11
HS-$PDMAEMA_8$-*b*- P*t*BA_2	92	1542	3.39	1922	1.16
HS-$PDMAEMA_8$-*b*- P*t*BA_4	91	1888	1.46	2280	1.15

[a] Determined from ^{1}H NMR. [b] Calculated by monomer conversion. [c] Determined by GPC. $M_{n,th}$ is the theoretical molecular weight of chain polymers and star-like block copolymers. $M_{n,app}$ is the apparent molecular weight measured by GPC.

Table S4. Compositions, molecular weights and molecular-weight distributions of star-like block copolymers.

Structure	conv(%)	g [a)]	$M_{n,star}$ [b)] (g/mol)	PDI	Arm number [b)]
4-Star-PtBA$_1$-*b*-PDMAEMA$_4$	85	0.63	4296	1.25	4.38
4-Star-PtBA$_2$-*b*-PDMAEMA$_8$	84	0.63	8245	1.30	4.17
4-Star-PtBA$_4$-*b*-PDMAEMA$_8$	90	0.63	9737	1.18	4.20
8-Star-PtBA$_1$-*b*-PDMAEMA$_4$	89	0.34	8208	1.25	7.84
8-Star-PtBA$_2$-*b*-PDMAEMA$_8$	83	0.34	15366	1.25	7.47
8-Star-PtBA$_4$-*b*-PDMAEMA$_8$	86	0.34	16350	1.27	6.78
12-Star-PtBA$_1$-*b*-PDMAEMA$_4$	82	0.24	11890	1.19	11.25
12-Star-PtBA$_2$-*b*-PDMAEMA$_8$	79	0.24	20726	1.26	9.96
12-Star-PtBA$_4$-*b*-PDMAEMA$_8$	78	0.24	22222	1.30	9.13
4-Star-PDMAEMA$_4$-*b*-PtBA$_1$	90	0.63	4478	1.24	4.47
4-Star-PDMAEMA$_4$-*b*-PtBA$_2$	87	0.63	4892	1.22	4.37
4-Star-PDMAEMA$_8$-*b*-PtBA$_2$	85	0.63	8079	1.26	4.13
4-Star-PDMAEMA$_8$-*b*-PtBA$_4$	86	0.63	9633	1.20	4.10
8-Star-PDMAEMA$_4$-*b*-PtBA$_1$	88	0.34	8252	1.39	7.70
8-Star-PDMAEMA$_4$-*b*-PtBA$_2$	86	0.34	9197	1.31	7.76
8-Star-PDMAEMA$_8$-*b*-PtBA$_2$	84	0.34	15305	1.28	7.42
8-Star-PDMAEMA$_8$-*b*-PtBA$_4$	83	0.34	15941	1.30	6.53
12-Star-PDMAEMA$_4$-*b*-PtBA$_1$	82	0.24	12018	1.25	11.19
12-Star-PDMAEMA$_4$-*b*-PtBA$_2$	85	0.24	13849	1.24	11.64
12-Star-PDMAEMA$_8$-*b*-PtBA$_2$	79	0.24	20159	1.32	9.64
12-Star-PDMAEMA$_8$-*b*-PtBA$_4$	80	0.24	21251	1.29	8.61

[a)] Calculated by Equation 1. [b)] Determined by GPC and Equation 1 and 2. $M_{n,star}$ is the actual molecular weight of the star-like block copolymers.

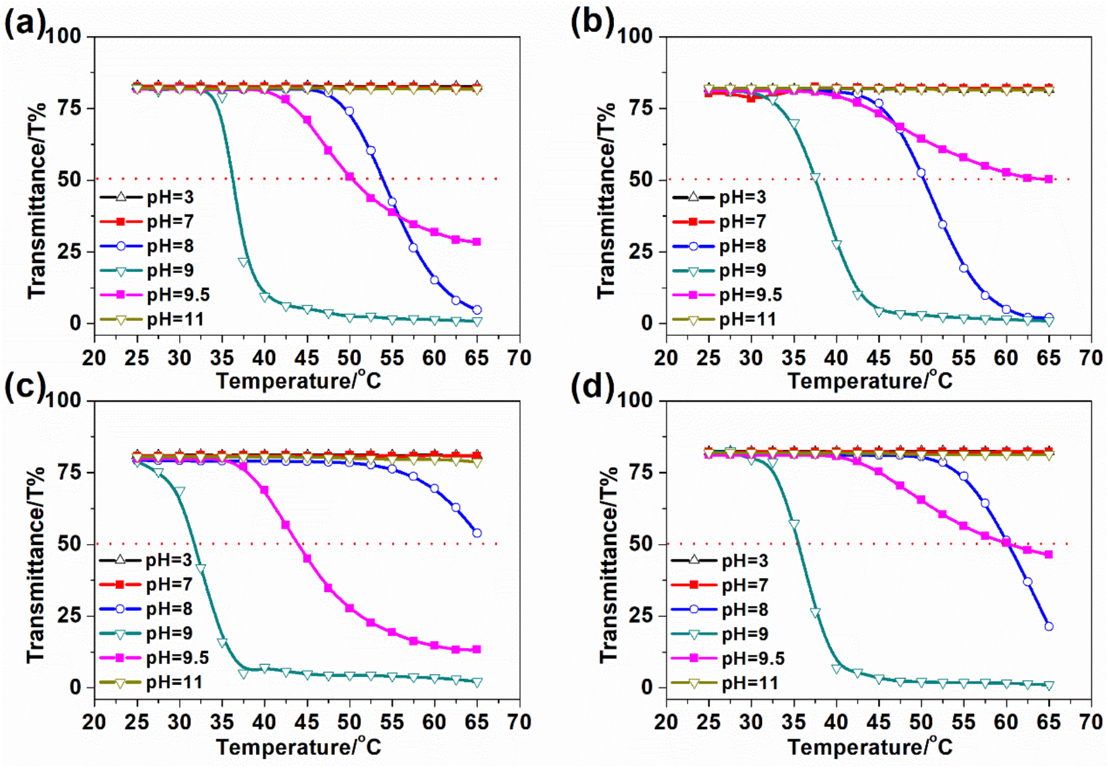

Figure. S10. The Effect of the pH value of 1.0 mg mL^{-1} aqueous solutions of 8-arm star-PAA$_1$-*b*-PDMAEMA$_4$ (a), 8-arm star-PAA$_2$-*b*-PDMAEMA$_4$ (b), 8-arm star-PDMAEMA$_4$-*b*-PAA$_1$ (c) and 8-arm star-PDMAEMA$_4$-*b*-PAA$_2$(d) on the LCST and temperature curve when the transmittance of the solution was monitored at 500 nm at a heating rate of 0.5°C min $^{-1}$.

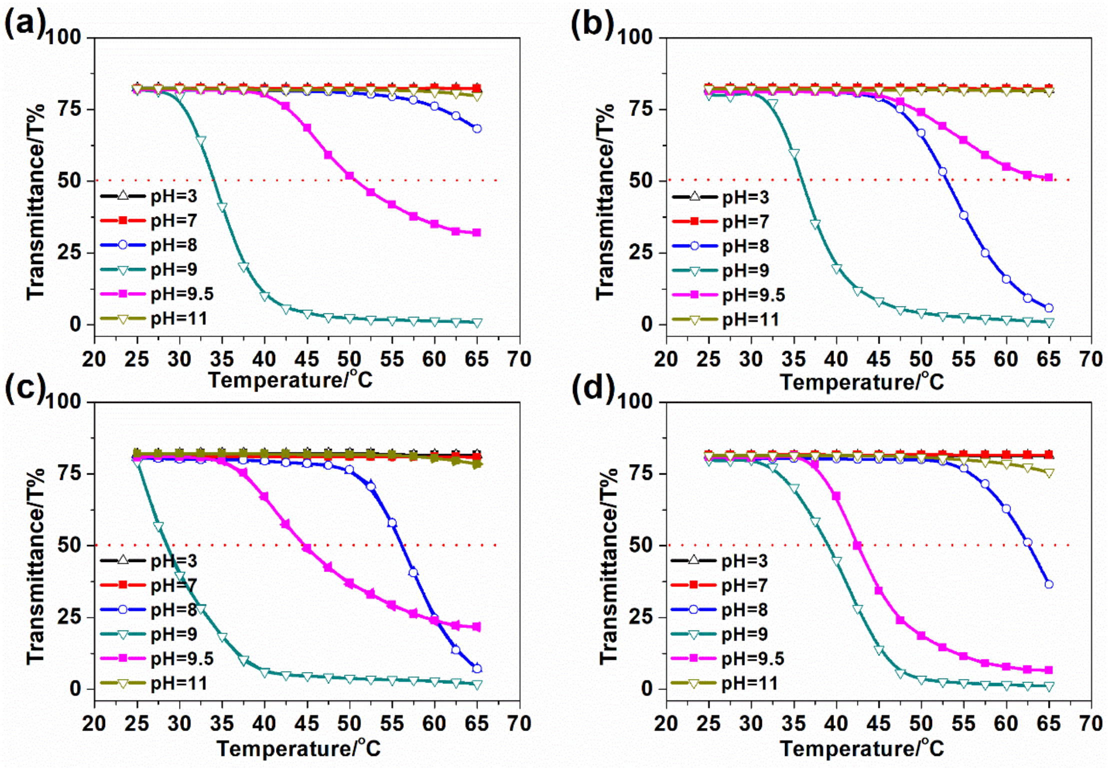

Figure. S11. The Effect of the pH value of 1.0 mg mL^{-1} aqueous solutions of 12-arm star-PAA$_1$-*b*-PDMAEMA$_4$ (a), 12-arm star-PAA$_2$-*b*-PDMAEMA$_4$ (b), 12-arm star-PDMAEMA$_4$-*b*-PAA$_1$(c) and 12-arm star-PDMAEMA$_4$-*b*-PAA$_2$ (d) on the LCST and temperature curve when the transmittance of the solution was monitored at 500 nm at a heating rate of 0.5^{o}C min $^{-1}$.

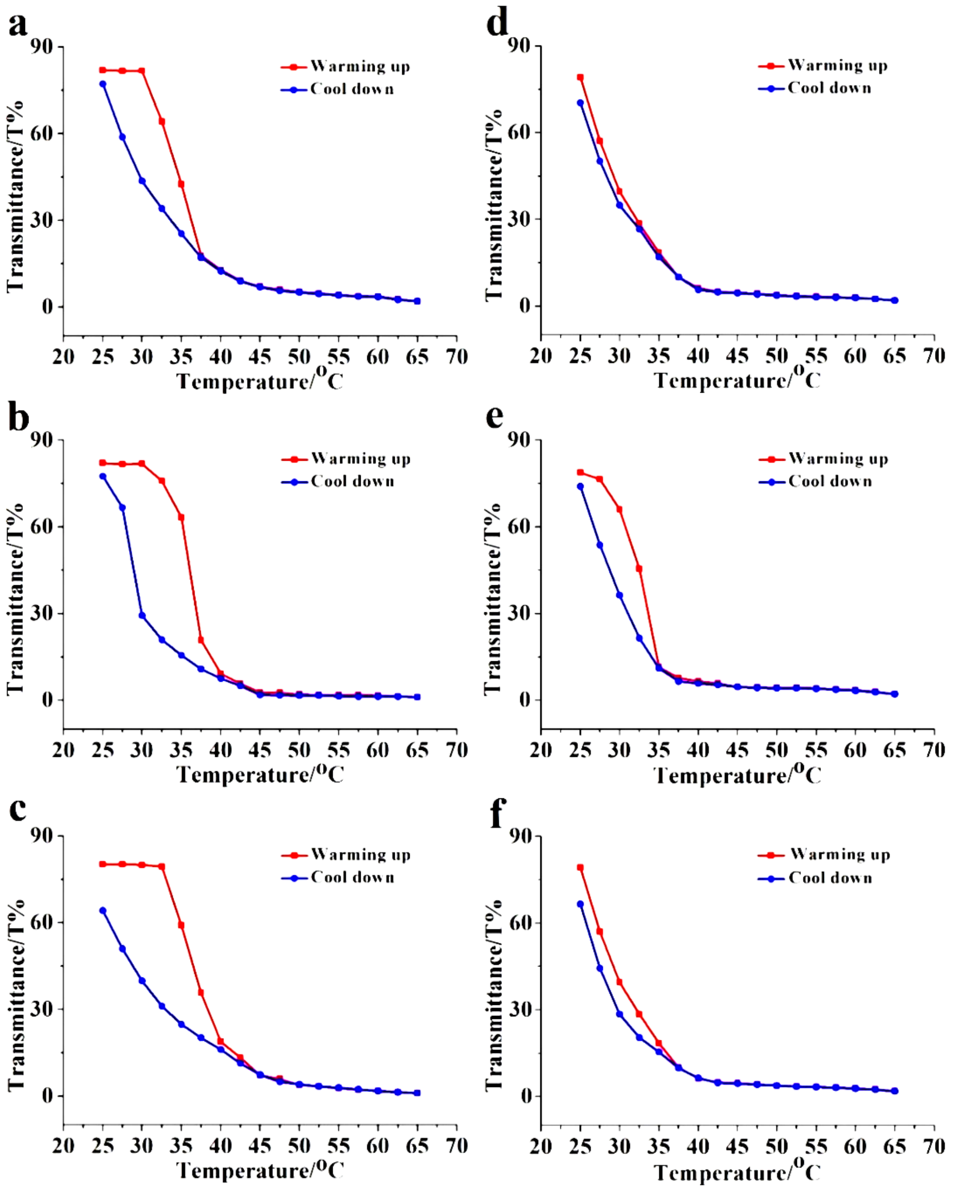

Figure. S12. Temperature dependences of the UV -visible transmittance of 1.0 mg mL^{-1} aqueous solutions at pH = 9.0 of 4-arm star-PAA$_2$-*b*-PDMAEMA$_4$ (a), 8-arm star-PAA$_2$-*b*-PDMAEMA$_4$ (b), 12-arm star-PAA$_2$-*b*-PDMAEMA$_4$ (c), 4-arm star-PDMAEMA$_4$-*b*-PAA$_2$ (d), 8-arm star-PDMAEMA$_4$-*b*-PAA$_2$ (e), 12-arm star-PDMAEMA$_4$-*b*-PAA$_2$ (f) on the LCST and temperature curve when the transmittance of the solution was monitored at 500 nm at a heating rate and cooling rate of 0.5°C min $^{-1}$.

The star-like block copolymers solution was heated and cooled successively, and the change curves of transmittance in the two processes were measured respectively. It can be seen from the Figure S11 that the thermo-sensitive stability of star-like block copolymers has a great relationship with their block sequence. When the PDMAEMA

segments are located at the end of the segment, the transmittance of the 4-arm, 8-arm and 12-arm star-like block copolymers in the cooling process is delayed compared with the change curve of the temperature increase transmittance, resulting in LCSTs decline occurs. When the PAA segments are located at the end of the segment, the transmittance curves of star-like block copolymers with all the number of arms are basically consistent with the transmittance curve at temperature rise, and there is no obvious delay phenomenon. This is related to the dissolved state of the polymers chains in solution. When the polymers solution is in an alkaline environment, the PDMAEMA segment at the end of the chain segment in star-PAA-*b*-PDMAEMA exhibit the hydrophobic shrinkage state. During the process of gradually dissolving the polymers as the temperature drops, their dispersion speed is slow, resulting in delay phenomenon. For star-PDMAEMA-*b*-PAA, the PAA segments located at the end of the segment is in the hydrophilic stretched state. During the temperature drop, the polymers disperse along with the dissolution, so there is no obvious delay.

43. Ferguson, C. J.; Hughes, R. J.; Pham, B. T. T.; Hawkett, B. S.; Gilbert, R. G.; Serelis, A. K.; Such, C. H., Effective ab Initio Emulsion Polymerization under RAFT Control. *Macromolecules* **2002,** *35* (25), 9243-9245.
